# Diversity of Interneurons in the Dorsal Striatum Revealed by Single-Cell RNA Sequencing and PatchSeq

**DOI:** 10.1016/j.celrep.2018.07.053

**Published:** 2018-08-24

**Authors:** Ana B. Muñoz-Manchado, Carolina Bengtsson Gonzales, Amit Zeisel, Hermany Munguba, Bo Bekkouche, Nathan G. Skene, Peter Lönnerberg, Jesper Ryge, Kenneth D. Harris, Sten Linnarsson, Jens Hjerling-Leffler

**Affiliations:** 1 Laboratory of Molecular Neurobiology, Department Medical Biochemistry and Biophysics, Karolinska Institutet, 17177 Stockholm, Sweden; 2 UCL Institute of Neurology, Queen Square, London WC1N 3BG, UK; 3 Brain Mind Institute, Ecole Polytechnique Federale de Lausanne, Lausanne, Switzerland; 4 UCL Department of Neuroscience, Physiology and Pharmacology, 21 University Street, London WC1E 6DE, UK

**Keywords:** striatum, interneuron, cell classes, single-cell RNA sequencing, Pthlh, parvalbumin, fast-spiking neurons, cortex, electrophysiology

## Abstract

Striatal locally projecting neurons, or interneurons, act on nearby circuits and shape functional output to the rest of the basal ganglia. We performed single-cell RNA sequencing of striatal cells enriching for interneurons. We find seven discrete interneuron types, six of which are GABAergic. In addition to providing specific markers for the populations previously described, including those expressing *Sst*/*Npy*, *Th*, *Npy* without *Sst*, and *Chat*, we identify two small populations of cells expressing *Cck* with or without *Vip*. Surprisingly, the *Pvalb*-expressing cells do not constitute a discrete cluster but rather are part of a larger group of cells expressing *Pthlh* with a spatial gradient of *Pvalb* expression. Using PatchSeq, we show that *Pthlh* cells exhibit a continuum of electrophysiological properties correlated with expression of *Pvalb*. Furthermore, we find significant molecular differences that correlate with differences in electrophysiological properties between *Pvalb*-expressing cells of the striatum and those of the cortex.

## Highlights

• Single-cell RNA sequencing of striatal interneurons reveals seven main classes
• Subclass-specific latent factor analysis reveals gradients of gene expression
• *Pvalb* neurons are not a discrete class but belong to a class expressing *Pthlh*
• Interneuron classes vary in their similarity to counterparts from cortex/hippocampus

## Graphical Abstract

**Figure:**
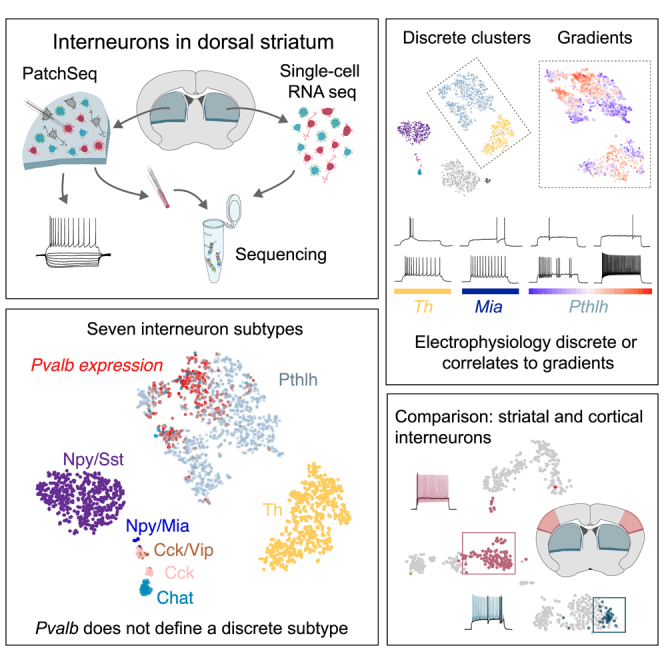

## Teaser

Muñoz-Manchado et al. describe the molecular and electrophysiological diversity of interneurons from the striatum. They find that in contrast to many other brain regions, cells expressing the gene *Pvalb* are not a discrete class of cells. This affects the interpretation of studies on striatal networks and their function.

## Introduction

The striatum is a predominantly inhibitory structure and acts as the main input structure for the basal ganglia system, as well as a plasticity center for reinforcement-based learning (Graybiel and Grafton, 2015, Kreitzer and Malenka, 2008, Sjulson et al., 2018). The influence of the diverse locally projecting interneurons in behavior and disease is less clear compared to the better-characterized principal neurons, the spiny projection neurons (SPNs, also known as medium spiny neurons) (Silberberg and Bolam, 2015, Tepper et al., 2010). Modern molecular tools are increasingly used to pinpoint a cell type-specific contribution to striatal-related disease (Girasole et al., 2018, Rapanelli et al., 2017, Skene et al., 2018). To correctly interpret the network effect, or behavioral outcome, of cell type-specific manipulation or to maximize the power of coupling genomics to disease, it is therefore crucial to understand the cell type composition of the striatum.

The interneurons of the striatum have been shown to make up around 5% of the total neuronal population (Graveland and DiFiglia, 1985). Most striatal interneurons signal via gamma-aminobutyric acid (GABA) to produce a local source of inhibition to their target cells. Studies trying to elucidate the cellular complexity traditionally rely on the expression of single markers coupled to electrophysiology (Gittis et al., 2010, Kawaguchi, 1993, Koós and Tepper, 1999, Kubota and Kawaguchi, 1994, Muñoz-Manchado et al., 2016). They have revealed a few principal GABAergic groups: *Pvalb*-expressing fast-spiking basket cells, *Sst/Npy*-expressing plateau low-threshold-spiking (pLTS) cells, *Calb2*-expressing cells, *Th*-expressing cells with heterogeneous firing patterns (Ibáñez-Sandoval et al., 2010), and a late-spiking *Npy*-positive, *Sst*-negative neurogliaform cell (NGC) (English et al., 2011, Ibáñez-Sandoval et al., 2011). In addition, the 5HT3a^EGFP^ mouse labels a large population of striatal interneurons that, together with the previously mentioned markers (with some overlap), make up 5% of the striatal neurons, arguing that we have mouse genetic tools to target all interneurons in the striatum (Muñoz-Manchado et al., 2016). Large-scale efforts using transcriptome sequencing of thousands of single cells in neuronal tissue hold promise to revolutionize our understanding of the neuronal diversity in the mammalian brain (Poulin et al., 2016). Previous striatal single-cell RNA sequencing (scRNA-seq) studies have focused on the SPNs, excluding interneurons from their analysis (Gokce et al., 2016).

Interneurons of the neocortex, hippocampus, and striatum share many properties. For example, *Pvalb*-expressing basket cells in all three structures exhibit dense local axonal arborization (Koós and Tepper, 1999), mainly targeting the cell body and proximal dendrites of principal cells (Kawaguchi et al., 1995, Kita, 1993). Electrophysiologically, they exhibit fast-spiking properties, subthreshold oscillations, high action potential (AP) threshold, short AP half-width, rapid and deep after hyperpolarization (AHP), and high maximum firing rate (Kawaguchi et al., 1995, Koós and Tepper, 1999). They both also have high connectivity probability with target cells (Planert et al., 2010) and are involved in feed-forward inhibition (Buzsáki, 1984, Gabernet et al., 2005, Szydlowski et al., 2013). Furthermore, striatal interneurons, along with most cortical and hippocampal interneurons, are derived from the same progenitor cells in the medial ganglion eminence (MGE) (Fishell and Rudy, 2011, Mayer et al., 2016).

Here we report an scRNA-seq analysis of striatal interneurons, combined with electrophysiology, to study how these modalities relate to one another. In addition to defining molecularly distinct classes, we describe gradients of gene expression within the clusters. We found that the striatal interneurons expressing *Pvalb* do not constitute a discrete class of cells but rather form part of a larger transcriptionally defined cluster expressing *Pthlh* (the gene encoding for parathyroid hormone-related protein) that also contains cells with low or no *Pvalb*. These Pthlh cells exhibited a broad continuum of intrinsic electrophysiological properties that correlated with *Pvalb* levels. Furthermore, we show by comparing striatal and cortical interneurons that there are large differences among striatal interneuron populations in the closeness to their cortical counterparts.

## Results

### scRNA-Seq of Interneurons of the Dorsolateral Striatum

Using fluorescence-activated cell sorting (FACS), we isolated cells from the dorsal striatum from either a 5HT3a^EGFP^ or a Lhx6^cre^::R26R-tdTomato mouse line labeling partly overlapping sets of striatal interneurons (data not shown). To achieve full coverage of the entire striatal neuronal population, we collected both fluorescently labeled and unlabeled cells for scRNA-seq using our previously described method (Zeisel et al., 2015) or fluorescent cells only using the STRT-seq-2i platform (Hochgerner et al., 2017). We will refer to these datasets as dataset A and dataset B, respectively.

**Figure 1:**
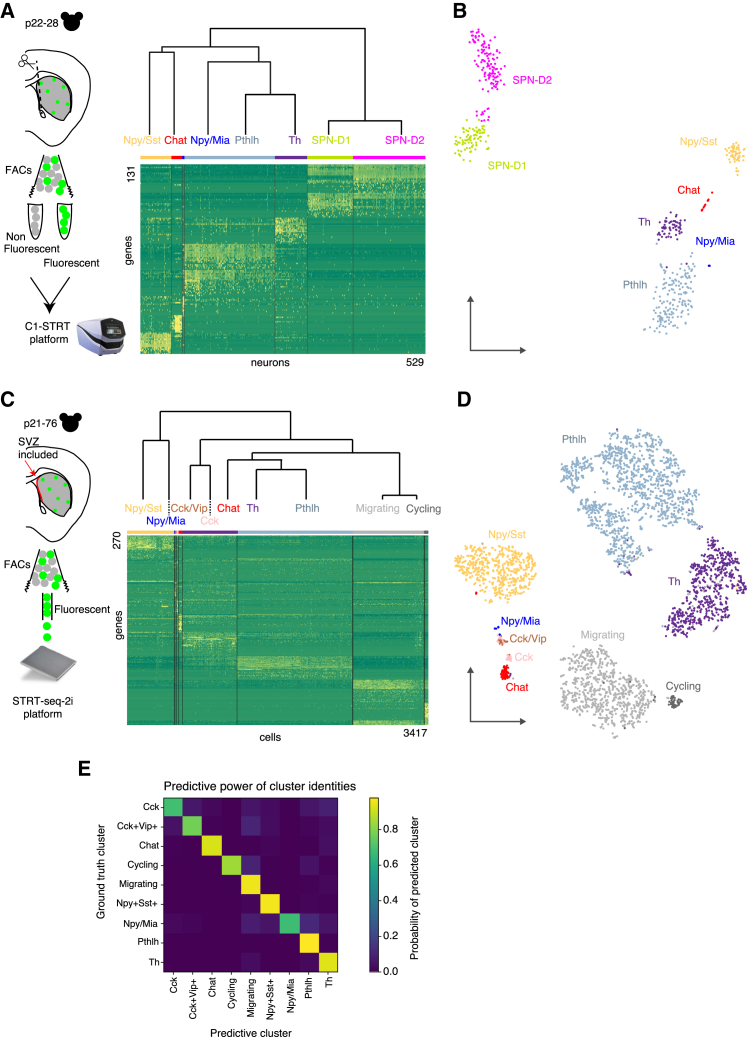
Single-Cell Sequencing Approaches in the Mouse Dorsal Striatum (A) Left: schematic representation of the experimental workflow (dataset A). Right: hierarchical analysis of neuronal clusters from dataset A. Below: heatmap showing the expression of the 131 most informative genes after BackSPIN analysis. (B) tSNE analysis of dataset A. (C) Left: schematic representation of the experimental workflow (dataset B). Right: hierarchical analysis of clusters obtained from dataset B. Below: heatmap showing the expression of the 270 most informative genes after BackSPIN analysis. (D) tSNE analysis of dataset B. (E) Predictive power of cluster identities using a trained random forest classifier on dataset B. See also Figure S1.

Dataset A contained 1,135 cells (passing quality control) from mice of postnatal day (P) 22–28 (approximately half were fluorescently labeled) (Figure S1A). We used the biclustering algorithm BackSPIN v.2 (Marques et al., 2016, Zeisel et al., 2015) to cluster cells and to identify the genes with the most specific expression patterns. To parse out cell identity not dependent on the activity state, for clustering only, we filtered out activity-dependent genes (Spiegel et al., 2014). We identified 529 cells as neuronal (Figure 1A) and 606 cells as non-neuronal (Figures S1B–S1D). Hierarchical clustering analysis (Figure 1A) revealed that the first split in the dendrogram gave one group of two clusters characterized by the expression of SPN markers such as *Ppp1r1b* (also known as Darpp-32) and *Bcl11b* (also known as Ctip2) and another group consisting of five clusters. These five clusters expressed high levels of either *Gad1* or *Chat*, suggesting that they were GABAergic or cholinergic interneurons, respectively. The clusters separated clearly when we visualized the data using t-distributed stochastic neighbor embedding (tSNE) (van der Maaten and Hinton, 2008) (Figure 1B).

**Figure 2:**
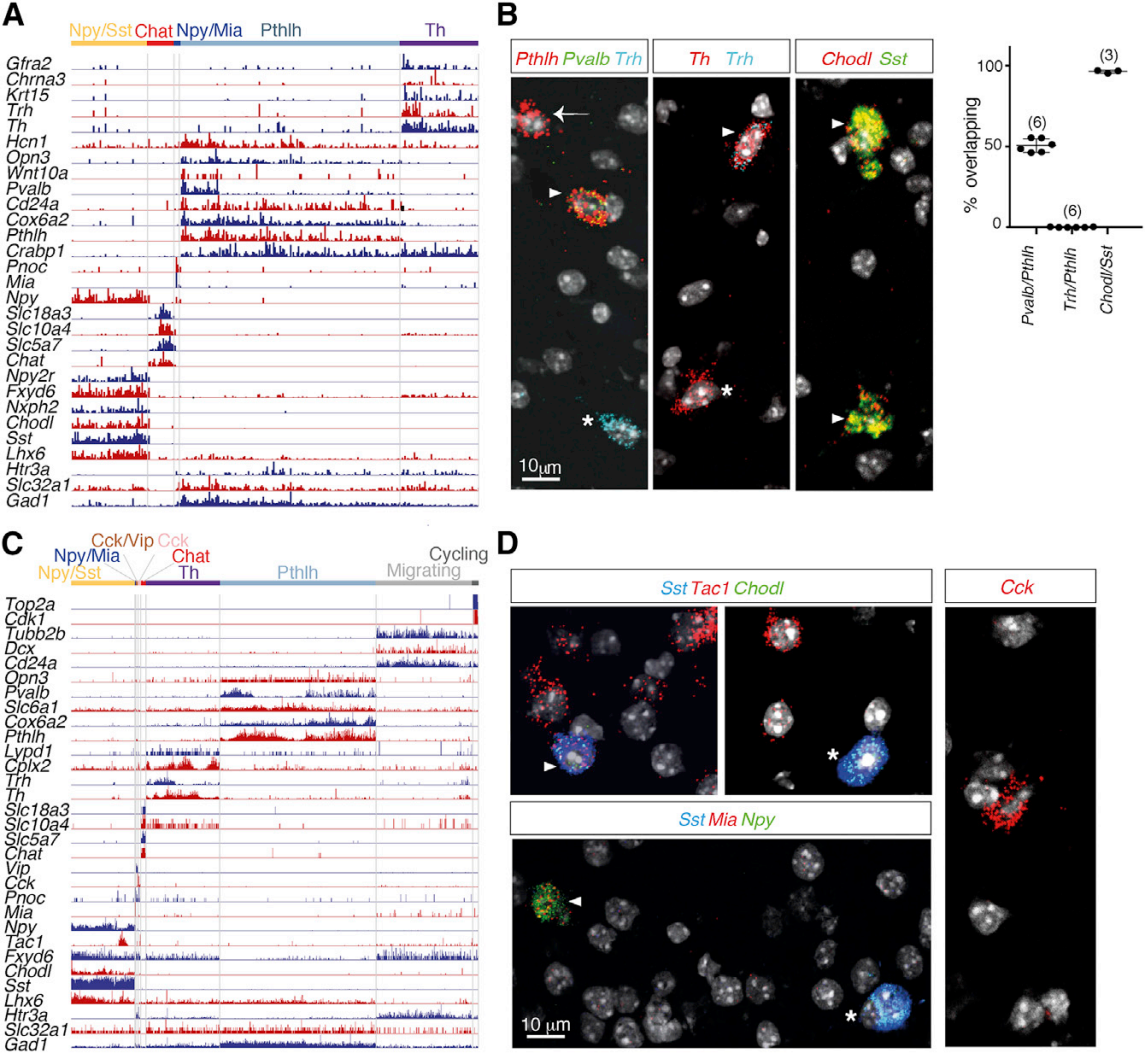
Characterization and Validation of the Striatal Interneuron Populations Obtained after Single-Cell Sequencing (A) Expression of main markers (molecular counts per individual cell) in dataset A. (B) Left: *in situ* hybridizations showing the co-expression of *Pthlh*, *Pvalb*, *Trh*, *Th*, *Chodl*, and *Sst* in the indicated combinations. Arrowheads show co-expression of *Pthlh* and *Pvalb*, *Th* and *Trh*, or *Chodl* and *Sst*. The arrow represents *Pthlh*-positive, *Pvalb*-negative cells, and stars represent *Trh*-positive cells and *Th*-positive, *Trh*-negative cells. Right: quantifications with the number of mice indicated in brackets (C) Expression of main markers (molecular counts per individual cell) in dataset B. (D) Representative *in situ* hybridization showing the co-expression of *Sst*, *Tac1*, *Chodl*, *Mia*, *Npy*, and *Cck* in the indicated combinations. Arrowheads indicate co-expression of either *Sst*, *Tac1*, and *Chodl* or *Npy* and *Mia*. Stars represent *Sst*-positive cells co-expressing either *Chodl* or *Npy*. Error bars represent mean ± SEM. See also Figure S2.

Dataset B included a broader range of mouse ages (P21–P26 and P55–P76) to also investigate age-dependent expression and contained 3,417 cells (Figures S1E–S1G). Performing the same cluster analysis as performed previously revealed nine clusters. Within the *Gad1*-expressing populations, in addition to the five interneuron clusters found in dataset A, we identified two small clusters expressing *Cck* alone or in combination with *Vip* (Figures 1C and 1D). Moreover, we defined a large cluster as migrating neuroblasts (expressing *Dcx*, *Tubb2b*, and *Cd24a*) and another was similar to the few cycling cells seen in dataset A. This was due to the inclusion of the subventricular zone (SVZ) (in dataset B only), which contains migrating neuroblasts also labeled in the 5HT3a^EGFP^ mouse (data not shown). All clusters in dataset B contained cells from both juveniles (P21–P26) and adults (P55–P76), demonstrating that these cell types were mature (Figure S1). Each interneuron class could be defined by either specific or combinatorial expression of markers as in dataset A (Figures 2A and 2C). Plotting known markers onto the tSNE analysis confirmed the identity of our clusters in both datasets (Figure S2). To validate the robustness of clustering, we performed an analysis using a random forest classifier. The average precision and recall were 97% and 96%, respectively, indicating a high level of separation of clusters. We then computed the probability, for each cluster, that its cells would be classified as any other type (Figure 1D).

### Striatal Interneuron Clusters and Subcluster Variability

The largest cluster of GABAergic interneurons in both datasets was a *Pthlh*-expressing population, also differentially expressing *Cox6a2* (cytochrome C oxidase subunit 6A2) and *Opn3* (opsin 3) (Figures 2A and 2C). *Pthlh* has been proposed as a marker for cortical *Pvalb*-expressing chandelier cells (Paul et al., 2017, Tasic et al., 2016). The Pthlh population contained all cells expressing high levels of *Pvalb* but also cells with low or no *Pvalb* expression. A manual quantification using *in situ* hybridization for *Pthlh* and *Pvalb* expression showed that the 50.88% ± 2.52% (n = 6 mice, P25, 1,390 cells) of the Pthlh population also expressed *Pvalb* (Figure 2B). This overlap was 63.5% ± 9.35% in tissue from 5 month mice (n = 3 mice, 349 cells), and we observed similar proportions of *Pvalb*^−^ Pthlh cells in scRNA-seq on young and adult mice, arguing that this does not depend on gradual maturation (Figure S1). However, performing *in situ* hybridization for Pvalb/Pthlh and immunohistochemistry for EGFP in Pvalb^cre^::RCE (Rosa26-CAG-EGFP) mice (Hippenmeyer et al., 2005) showed that a small proportion of Pthlh cells not expressing Pvalb were labeled (Figure S4). This argues that at least some *Pvalb*-negative Pthlh cells had at some point expressed *Pvalb* and that this expression could be influenced by cell-extrinsic mechanisms.

The second-largest GABAergic interneuron population was characterized by the expression of *Th*. We identified several additional Th cell-specific markers, including *Chrna3* and *Gfra2*. In both datasets, a proportion of *Th*-expressing neurons also expressed *Trh*, the gene for thyrotropin-releasing hormone (Figure 2A). In our scRNA-seq data, we also observed sporadic expression of *Th* outside the main Th group in the Pthlh and Npy/Sst class (Figures 2A and 2C), but little overlap (0.19% ± 0.12% in Pthlh cells; n = 3 mice, P25, 1,390 cells) was seen using *in situ* hybridization for *Pthlh* and *Trh* (Figure 2B).

For the Npy/Sst population (also expressing *Nos1*; data not shown), we identified the specific marker *Chodl* (Figures 2A and 2C) and confirmed this using *in situ* hybridization (96.18% ± 0.83% of *Sst*^+^ cells were also *Chodl*^+^; n = 3 mice, P25, 244 cells) (Figure 2B). *Chodl* is also expressed by *Nos1*-expressing cells in the cortex (Tasic et al., 2016). Additional markers that were exclusively expressed by the Npy/Sst group include *Nxph2*, a poorly characterized gene, and *Npy2r*, expressed by cortical *Vip*-expressing cells (Tasic et al., 2016, Zeisel et al., 2015). We also identified a subset of the Npy/Sst cells expressing *Tac1* (Figures 2C and 2D), but this, similar to *Trh* and *Pvalb*, did not emerge as a discrete cluster in the analysis.

In both datasets, we found a small distinct cluster of cells that were *Npy*^+^/*Sst*^−^, a marker combination previously shown to demarcate striatal NGCs (Ibáñez-Sandoval et al., 2011). They were characterized by their specific expression of *Mia*, a gene specifically expressed by a putative cortical *Npy*^+^/*Sst*^−^ NGC-type Int14 (Zeisel et al., 2015) (Figures 2A and 2C). We confirmed the striatal location of these cells using *in situ* hybridization for *Sst*, *Npy*, and *Mia* (Figure 2D). They also expressed *Car4* (data not shown), another marker for cortical NGCs (Niquille et al., 2018), but in this manuscript we refer to these cells as Npy/Mia cells. In dataset B, we found an additional small population of cells expressing *Cck* with or without *Vip* in the striatum. Using *in situ* hybridization for *Cck* (Figure 2D) we found sparse cells in the dorsal striatum. We confirmed the expression of the Cck protein using immunohistochemistry and found 48 cells in four hemispheres (12 cells/hemisphere; 64 sections each 50 μm thick were analyzed, n = 2 mice; data not shown). We could, similar to the signal in the Allen Brain Atlas, see a slightly higher density of Cck cells in the posterior dorsal striatum.

**Figure 3:**
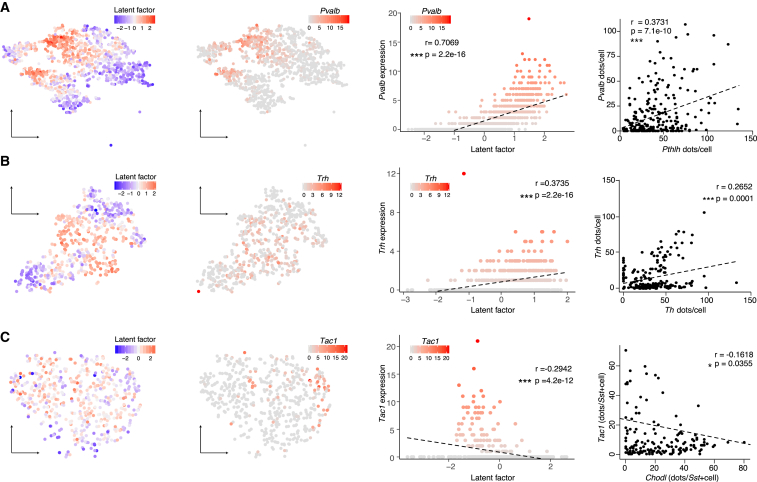
Latent Factor Correlates with Gradient-Wise Gene Expression Changes within Pthlh, Sst, and Th Populations (A) Left: tSNE of Pthlh population with the distribution of the latent factor or the expression of *Pvalb*. Right: scatterplot showing correlation of latent factor and *Pvalb* expression and the quantification of the *in situ* hybridization (dots/cell), correlating *Pthlh* and *Pvalb* (3 mice, 257 cells). (B) Left: tSNE of Sst population with the distribution of the latent factor or the expression of *Tac1*. Right: scatterplot showing correlation of latent factor and *Tac1* expression and the quantification of the *in situ* hybridization (dots/cell), correlating *Chodl* and *Tac1* within *Sst*+ cells (3 mice, 206 cells). (C) Left: tSNE of Th population with the distribution of the latent factor or the expression of *Trh*. Right: scatterplot showing correlation of latent factor and *Trh* expression and the quantification of the *in situ* hybridization (dots/cell), correlating *Trh* and *Th* (4 mice, 207 cells). Analyses were done on dataset B. r and p values were acquired using Pearson’s correlation. See also Table S1.

The BackSPIN algorithm would not further subdivide these clusters, but to reveal additional variability, in the form of gradients in the data, we applied a latent factor analysis (Harris et al., 2018) for larger clusters separately (Figure 3; Table S1). This unbiased analysis finds the multidimensional expression vector that explains the largest amount of variability in gene expression within the population analyzed. For Pthlh cells, the latent factor was highly correlated with the expression of *Pvalb* (latent factor score 1.7), showing that this gene is part of the largest transcriptional program along which these cells vary (Figure 3A). We found a similar gradient in the Th cells driven to a large extent by the expression of peptides (*Gal* and *Trh*) but also *Rgs4* and *Nnat* (Figure 3B). We could confirm the gradient observed in scRNA-seq using quantitative *in situ* hybridization for *Pvalb* and *Trh* (Figures 3A and 3B). For the Npy/Sst cells, the latent factor analysis did not reveal an equally strong gradient, but among the genes carrying the most weight were those peptides that we had identified manually. On one side were *Rbp4*, a gene also expressed by layer 5 pyramidal cells (Harris et al., 2014), and *Tac1*, which is a precursor protein for Neurkinin A and Substance P, while ubiquitous cell type markers *Npy*, *Sst*, and *Chodl* were on the other side. We confirmed differential expression of *Tac1* within the Npy/Sst cluster, which was anticorrelated to the ubiquitous *Chodl* expression when investigated in tissue (r = −0.162, p = 0.0355) (Figure 3C).

### The Pthlh Interneurons Exist in a Transcriptional Gradient that Is Spatially Organized

**Figure 4:**
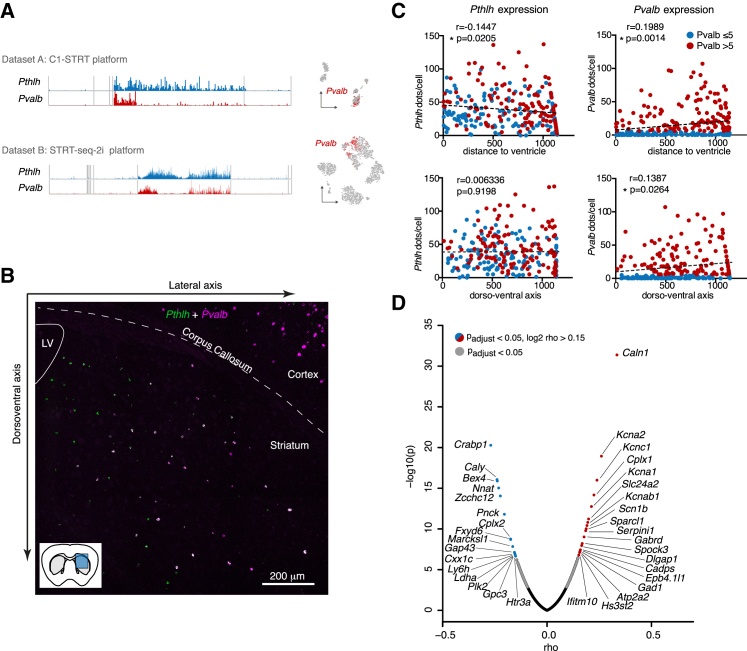
The *Pthlh* Population: *Pvalb* Co-expressing Cells and Spatial Organization (A) Molecular counts of *Pthlh* and *Pvalb* in each cell from BackSPIN analysis in both datasets, along with *Pvalb* expression labeled in red on tSNE plots. (B) Representative *in situ* hybridization showing the distribution in the dorsoventral and mediolateral axis of *Pthlh* and *Pvalb* expression. (C) Correlation of *Pthlh* and *Pvalb* expression with mediolateral or dorsoventral axis cell position. r and p values were acquired using Pearson’s correlation. (D) Volcano plot of genes with anti-correlation (blue) or correlation (red) to *Pvalb*. The y axis represents the log10 adjusted p value, and the x axis shows the rho value, both acquired using Spearman’s rank correlation coefficient. See also Figure S3 and Table S2.

In both datasets, we found that only a proportion of Pthlh cells expressed robust levels of *Pvalb* (Figure 4A) and that this was the major contributor to the latent factor. Quantitative fluorescence *in situ* hybridization in tissue (n = 3 mice, 257 cells) did not reveal discrete patterns but rather pointed at a continuum of expression (Figures 3A, 4B, and 4C; channels in Figure 4B are split in Figure S5). Furthermore, *Pvalb* expression in the Pthlh population was positively correlated with the distance to the lateral ventricle (mediolateral axis) and with the dorsoventral axis (r = 0.1989, p = 0.0014 and r = 0.1387, p = 0.0264, respectively) (Figure 4C). This was likely not due to the size of the cells, leading to a higher detection rate, because we observed the opposite for *Pthlh* (r = −0.1447, p = 0.0205 and r = 0.006336, p = 0.9198, respectively). The latent factor reveals a larger overall structure; to investigate whether *Pvalb* expression was correlated directly with a transcriptional program, we performed Spearman’s correlation test. We found that 19 genes were significantly positively correlated with *Pvalb* within the Pthlh population (rho > 0.15 and adjusted p value < 0.05) while 16 were anti-correlated (rho < −0.15 and adjusted p value < 0.05) (n = 12 mice, 1,313 cells) (Figure 4D; Table S2). The highest correlated gene was *Caln1*, a calmodulin-like calcium sensor (Wu et al., 2001), and three of the five most positively correlated genes were voltage-gated potassium channels necessary for the fast-spiking phenotype of Pvalb cells (*Kcna2*, *Kcnc1*, and *Kcna1*). A gene ontology (GO)-term analysis revealed that in addition to voltage-gated potassium channels, the most prominent features were calcium sensitivity and metabolic activity also coupled to the increased energy expenditure of frequently firing cells (data not shown).

### Electrophysiological Properties within the Pthlh Population

**Figure 5:**
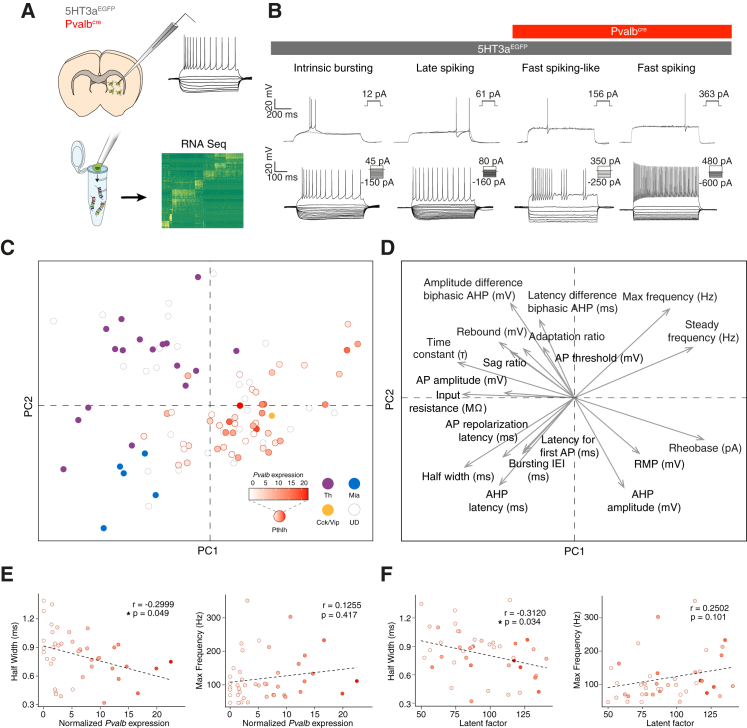
Electrophysiological Properties within the *Pthlh* Population Revealed Using PatchSeq (A) Schematic of the PatchSeq protocol. (B) Representative traces of electrophysiological subtypes detected in the mouse lines used for the recordings. (C) Principal-component analysis (PCA) based on 19 electrophysiological parameters from 98 interneurons. The color of the dots refers to the molecular identity acquired by mapping them onto BackSPIN clustering of dataset B. (D) Vector factor map analysis showing how (arrow size) the parameters contribute to (A). (E) Correlations of AP half-width and maximum frequency with normalized *Pvalb* expression within the Pthlh population. (F) Correlations of AP half-width and maximum frequency with the Pthlh cell latent factor. See also Figures S4 and S5 and Table S3.

Given that striatal *Pvalb*-expressing neurons have previously been shown to be fast-spiking basket cells (Koós and Tepper, 1999), we set out to investigate how electrophysiological properties varies within the Pthlh population in the dorsolateral striatum. To this end, we performed PatchSeq analysis of neurons labeled in the 5HT3a^EGFP^ and *Pvalb*^cre^::RCE/tdTomato mouse lines (Figure 5A). These lines together allow us to survey a larger proportion of the Pthlh population than we could with the *Pvalb*^cre^ line alone, but they include the Th, Npy/Mia, Cck, and Cck/Vip populations (Muñoz-Manchado et al., 2016). We sequenced 144 recorded cells, out of which 98 cells passed our quality-control thresholds (Figure S4). We observed several key electrophysiological features previously described for striatal interneuron classes (Figure 5B). In addition, some cells exhibited hallmarks of fast-spiking cells (short AHP latency, subthreshold oscillation, and high AP threshold), but not high-frequency firing. We called these fast-spiking-like cells (Figure 5B).

Individual transcriptomes were mapped onto the clustering from dataset B using a bootstrapping algorithm, allowing all seven interneuron classes as possible outcomes. We classified the identity of cells mapped to a specific cell type with p < 0.05 (70/98 cells). No cell was associated with a class not expected to be labeled by the mouse lines used (i.e., Sst/Npy or Chat cells) (Figure S6). Plotting the transcriptional identity (and normalized expression of *Pvalb* as a color code) onto a principal-component analysis (PCA) of 19 electrophysiological parameters (Figures 5C and 5D; Figure S7; Table S3) showed that the *Th*-expressing neurons were distinct along one axis while the electrophysiological properties of the Npy/Mia and Pthlh populations seemed to exist in a continuum along a perpendicular axis. These axes both depend on a mix between first and second principal components (PC1 and PC2), with the first axis mainly influenced by parameters generally related to low firing threshold and bursting behavior of neurons (Figure 5D). The second axis was driven by parameters related to the fast-firing properties of neurons. In the continuum along the second axis, the Npy/Mia cells were at one far end while the Pthlh cells occupied a larger area. Npy/Mia cells are putative late-spiking NGCs, and we could clearly observe the typical firing patterns in these cells (late spiking in Figure 5B). The Pthlh cells expressing high levels of *Pvalb* were spread throughout the Pthlh group, suggesting that *Pvalb*-low and *Pvalb*-high cells cannot reliably be identified based on intrinsic electrophysiological properties in the dorsolateral striatum. However, plotting normalized *Pvalb* gene expression against two electrophysiological parameters typically identifying fast-spiking cells (AP half-width and maximum [max] firing frequency) revealed correlations in the expected directions but only reached significance for AP half-width (r = −0.2999, p = 0.049) (Figure 5E). Both parameters were more correlated with the latent factor of Pthlh (AP half-width, r = −0.3120, p = 0.034; maximum firing frequency, r = 0.2502, p = 0.101) (Figure 5F) than with *Pvalb* alone. This is in accordance with our finding that several genes necessary for a fast-spiking phenotype correlated with *Pvalb*.

### Striatal and Cortical *Pvalb* Cells Are Transcriptionally Different

**Figure 6:**
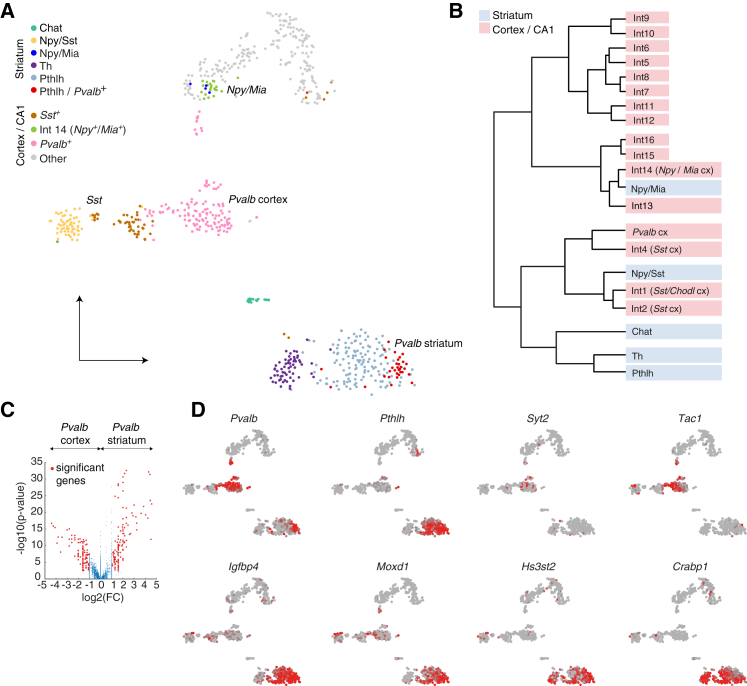
Comparing Single-Cell Transcriptomes of Interneurons from the Striatum and Cortex and Hippocampus (A) tSNE visualizations of the individual transcriptome of striatal (dataset A) and cortical-hippocampal interneurons. (B) Hierarchical clustering of the same cell populations shown in (A). (C) Differential gene expression analysis of striatal *Pvalb*-positive cells and their cortical counterparts. The y axis represents the log10 (p value) acquired using Wilcoxon rank-sum test, and the x axis shows the log2 (fold change). Significantly differentially expressed genes are marked in red. (D) tSNE plots showing examples of differentially expressed genes in *Pvalb*^+^ cells in striatum and cortex and hippocampus. See also Table S4.

To investigate how the transcriptional profiles of developmentally related interneuron classes differ after integration into different kinds of networks, we compared the expression profiles of striatal interneurons from dataset A (Figure 1A) to those of interneurons from the primary somatosensory (S1) cortex and CA1 hippocampus (Figure 6). This mixed cortex and hippocampus (cx/hc) interneuron dataset includes cells from a previous study (Zeisel et al., 2015) supplemented with cells from the S1 of *Pvalb*^cre^ mice procured using the same pipeline. A tSNE plot showed that within the cx/hc interneurons, the caudal ganglionic eminence (CGE)-derived interneurons (non-*Sst*/non-*Pvalb*-expressing) (gray- and light green-labeled cells, Figure 6A) are clearly distinct from the MGE-derived *Pvalb*- and *Sst*-expressing cells. Striatal interneurons, although also MGE derived, were distinct from MGE-derived cx/hc interneurons but to a different extent depending on subtype. The striatal Npy/Mia cells were intermingled with cx/hc Int14 (also expressing *Npy* and *Mia*). Striatal Npy/Sst cells clustered separately but still relatively close to their cx/hc counterparts. As suggested by the expression of *Chodl*, striatal Npy/Sst cells were most closely located to the *Chodl*-expressing *Sst*^*+*^ cx/hc interneurons (the small cluster of brown cells between striatal Npy/Sst and cx/hc *Sst*^*+*^ cells; data not shown). Finally, the striatal Th and Pthlh populations clustered together distinct from any cx/hc counterparts. These relationships were supported by a hierarchical cluster analysis (Figure 6B).

A differential expression analysis between striatal Pthlh/*Pvalb*-high cells and *Pvalb*-expressing cells from the cortex revealed 246 or 412 genes that were significantly higher or lower, respectively (p_adjust_ ≤ 0.05, fold change ≥ 2) in striatum compared to cortex (n = 12 mice, 112 cortical cells and 34 striatal cells) (Figure 6C; Table S4). We plotted the expression of eight markers that were significantly different onto the tSNE plot to showcase genes that can be used as putative markers for the two populations (Figure 6D). The differences include channels necessary for fast firing such as *Kcna1* and *Kcnc1* and mitochondrial genes *Cox6a2*. As suggested by *Pthlh* expression, we observed differences in expression of genes encoding for signaling peptides (*Pthlh* in striatum and *Cck* and *Npy* in cortex) among the top 6 genes enriched for each cell type. We also found higher striatal expression of *Hapln1* and *Adamst5*, which are involved in the formation and maintenance of perineuronal nets (PNNs) (Dubey et al., 2017, Giamanco et al., 2010). The most significant GO term for both lists was Synapse (data not shown), and this includes several genes shown to be important for synaptic plasticity (*Pcp4*, *Epha4*, and *Nxph1* in striatal cells and *Snca* and *Synpr* in cortical cells), suggesting that perhaps there are synaptic specializations or forms of synaptic plasticity in each of the cell types. This has been observed among cortical interneuron classes (Paul et al., 2017).

### Parvalbumin-Expressing Interneurons from Striatum and Cortex Show Different Intrinsic Electrophysiological Properties

**Figure 7:**
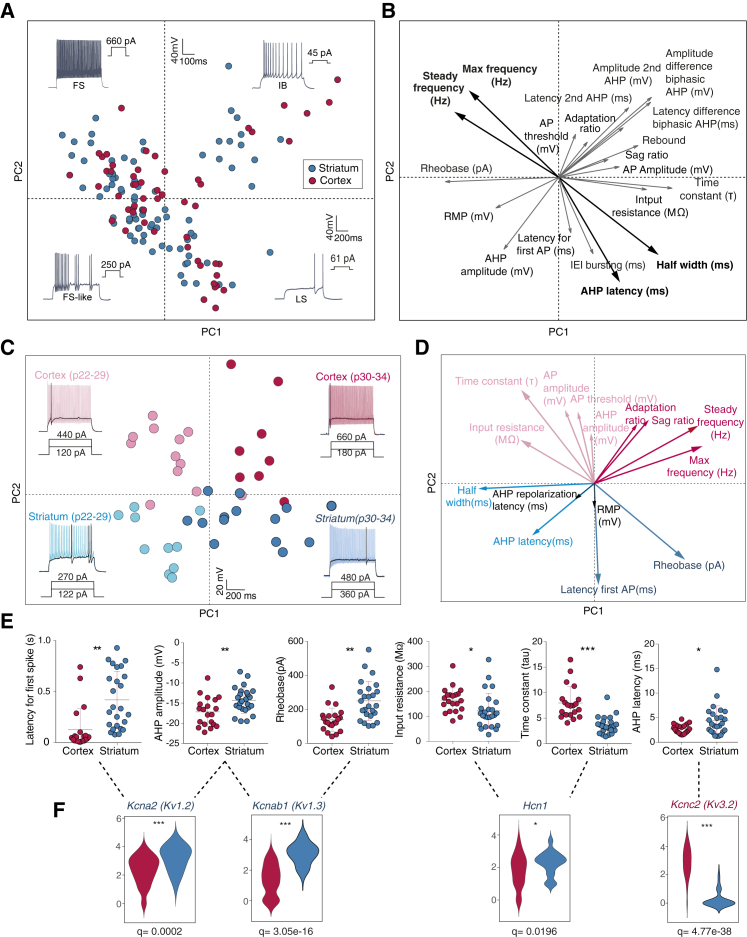
*Pvalb*-Expressing Interneurons from Striatum and Cortex Exhibit Distinct Intrinsic Properties that Match with Gene Expression (A) PCA using 19 electrophysiological parameters acquired from recordings of 135 cortical and striatal interneurons using *Pvalb*^cre^ and 5HT3a^EGFP^ mouse lines. (B) Vector factor map analysis showing the parameters used in the PCA in (A) and their contribution (arrow size). (C) PCA based on 15 electrophysiological parameters obtained from *Pvalb*^cre+^ cells recorded in layer 5 S1 cortex and dorsolateral striatum. Representative traces are shown in each quarter. (D) Vector factor map analysis showing the parameters used for the PCA in (A) and their contribution (arrow size). (E) Selected parameters that were significantly different between cortical (20) and striatal (25) Pvalb^cre+^ cells, tested using unpaired t test Benjamini-Hochberg correction for multiple testing (Table S5). Error bars represent mean ± SEM. (F) Expression of selected differentially expressed ion channels involved in fast-spiking behavior in Pvalb^cre+^ cells. Differential expression was tested upon log transforming the data, using unpaired t test with Benjamini-Hochberg multiple testing correction (Figure S6). FS, fast spiking; FS-like, fast spiking like; IB, intrinsic bursting; LS, late spiking; BiaRS, biphasic AHP regular spiking. See also Figures S6 and S7 and Table S5.

Striatal and cx/hc *Pvalb*-expressing cells are generally considered electrophysiologically similar, with a few reports on differences in individual properties (Kaiser et al., 2016). In light of the molecular differences, we performed whole-cell patch-clamp recordings of 135 cortical and striatal cells from cortex layer V and dorsolateral striatum in *Pvalb*^cre^ mice, as well as cortex layer II/III and dorsolateral striatum in 5HT3a^EGFP^ mice. Again, the first two PCs revealed one diagonal axis, which corresponded to fast-spiking properties (e.g., maximum frequency and AP half-width) and a perpendicular axis corresponding to bursting and low-threshold firing (Figures 7A and 7B). A hierarchical cluster analysis revealed that striatal and cortical fast-spiking cells, as well as bursting and/or low-threshold neurons, separated early, whereas late-spiking cells did not (Figure S6). A PCA of the *Pvalb*^cre+^ cells alone (Figures 7C and 7D) revealed that the age of the animal contributes to the first principal component (PC1, mainly fast firing) and that the difference between cortical and striatal neurons constituted the PC2 (mainly due to delay to the first AP and time constant). Several variables were also significantly different in a pairwise comparison (n = 8 mice, 20 cortical cells and 25 striatal cells) (Figure 7E; Table S5). Although striatal *Pvalb*^cre+^ cells had a shorter time constant and lower input resistance, they had higher rheobase, longer latency to first spike at rheobase, and higher AHP amplitude and latency. These differences in electrophysiological properties were accompanied by significant differences in ion channels involved in fast-spiking behavior (n = 12 mice, 122 cortical cells and 34 striatal cells) (Figure 7F; Figure S7). The higher expression of *Kcna2* (Kv1.2), *Kcnab1* (Kv.1.3), and *Hcn1* detected in striatal *Pvalb*-expressing cells compared to their cortical counterparts can explain their latency to first spike (delayed firing) (*Kcna2*) (Shen et al., 2004), reduced AHP amplitude (*Kcna2/Kcnab1*) (Erisir et al., 1999, Wang et al., 2013), increased rheobase (*Kcnab1*) (Kasten et al., 2007), and lower input resistance and membrane time constant (*Hcn1*) (Aponte et al., 2006, Huang et al., 2009). *Kcnc2* (Kv3.2), which promotes sustained fast spiking by allowing a rapid repolarization after AP discharge (Erisir et al., 1999), was found to be higher expressed in cortical *Pvalb*^cre+^ cells. This shows that despite being grossly similar, there are significant differences between the electrophysiological intrinsic properties of cortical and those of striatal *Pvalb*^cre+^-labeled interneurons that can be corroborated by transcriptional analysis.

## Discussion

In this study, we have performed scRNA-seq and electrophysiological investigation of striatal interneurons. We provide an extensive list of markers for all striatal cell types, including six discrete classes of GABAergic interneurons and one cholinergic class of interneurons. These six classes included one class of interneurons expressing *Pthlh* that contained the *Pvalb*-expressing interneurons but also a significant proportion lacking *Pvalb*. The *Pvalb* expression within the Pthlh population existed on a transcriptional gradient correlating with a dorsomedial to ventrolateral axis and is correlated to the fast-spiking electrophysiological properties.

We observed a molecular diversity that was considerably lower than observed in cx/hc using similar clustering methods and sampling depth to dataset A (Tasic et al., 2016, Zeisel et al., 2015). Furthermore, with more cell sampled (albeit at a lower depth), we could identify additional smaller clusters due to sampling of rare cell types, but the main clusters did not split into more subtypes. In dataset B, we analyzed 3,417 cells, out of which 619 were Th cells. Stereological analysis has shown that each hemisphere contains 2,756 ± 192.4 Th-expressing neurons (Unal et al., 2011). We thus have in our study sampled roughly 20% of this population. It is possible that we would detect additional clusters with deeper sampling (number of cells or sequencing depth), as has been shown in hippocampal interneurons (Harris et al., 2018). However, striatal SPNs have been shown to exhibit gradients of gene expression not captured by clustering (Gokce et al., 2016). This is in line with the substantial additional structure observed in our data, beyond the clusters, detected using latent factor analysis. It is possible that cellular diversity in the striatum is arranged in a less discrete manner compared to the cx/hc and that the continua we observed could be further divided into subclasses by using more sensitive clustering algorithms.

Our findings suggest not that *Pvalb*-expressing interneurons are a discrete class in the striatum but rather that *Pthlh* is a better marker for this larger class of interneurons. Although cells within the Pthlh class shared some electrophysiological characteristics typical of fast-spiking interneurons, we observed a substantial electrophysiological continuum within this group. The slowest-spiking Pthlh cells of the fast-spiking-like type (some of which clearly expressed *Pvalb*) were close in PCA space, forming a continuum with late-spiking putative NGCs. This looked different for isocortical fast-spiking neurons, for which there was a clearer separation in PCA space from cortical NGCs.

We could correlate AP half-width, perhaps the most reliable marker of a fast-spiking phenotype, to both *Pvalb* expression and the latent factor for Pthlh cells. There have been reports of fewer fast-spiking units and weaker gamma-rhythm power (which depends on the activity of fast-spiking units), in *in vivo* recordings of ventromedial striatum in the rat (Berke et al., 2004). This has been proposed to be due to lower amounts of fast-spiking basket cells in that area, as determined by Pvalb immunohistochemistry (Gerfen et al., 1985). Our data suggest that although there are lower levels of *Pvalb* medially, this is not due to an absence of *Pthlh*-expressing cells. This suggests that by using Pvalb-cre mice to study fast-spiking cells in the dorsomedial striatum, researchers might significantly underestimate the functional role of the Pthlh cells.

Two morphological types of striatal fast-spiking interneurons have been reported (Koós and Tepper, 1999), and these could correspond to our Pvalb high and low populations. It has been shown that parvalbuminergic striatal fast-spiking cells from lateral and medial striatum have different (but partly overlapping) electrophysiological properties and that they receive differential input from cortical areas (Monteiro et al., 2018). Our data suggest that these two reported types exist on a molecular continuum rather as two separate states. The weaker correlation that we observed in our PatchSeq experiments could perhaps be explained by our record of neurons predominantly in the dorsolateral part of the striatum. Furthermore, two populations of *Pvalb* cells distinguished by the expression of *Scgn* have been described (Garas et al., 2016, Kosaka et al., 2017). We only observed a few cells expressing *Scgn* in dataset B (within the Cck, Chat, and Pthlh populations), but these did not cluster together.

Work using reporter mice for *Th* has shown considerable electrophysiological heterogeneity within these groups of neurons (Ibáñez-Sandoval et al., 2010, Muñoz-Manchado et al., 2016). We only observed a single molecular *Th*-expressing cluster. Nonetheless, we found spurious expression of *Th* in individual cells within other clusters. This expression is not enough to cluster these cells separately, but if reflected in the *Th*^EGFP^ mouse, it might contribute to an increased level of electrophysiological diversity.

We observed two clusters expressing *Cck* with or without *Vip*. Both *Cck*- and *Vip*-expressing populations have been described in the striatum of the rat (Hökfelt et al., 1988, Takagi et al., 1984, Theriault and Landis, 1987). We confirmed their existence on both the mRNA and the protein level, but these were sparse and could potentially represent misguided Cck cells heading for cortical structures. Striatal Cck cells have been proposed to perhaps also express *Th* (Tepper et al., 2010), but we saw no evidence of this in our data.

Striatal Pthlh (which includes *Pvalb*^*+*^) cells were molecularly distant from the cortical *Pvalb*- and *Sst*-expressing cells. All these classes are MGE derived and have been shown to be clonally related (Mayer et al., 2016). *Pvalb*-expressing neurons in the cortex have been shown to tune their intrinsic electrophysiological properties in response to activation (Dehorter et al., 2015, Donato et al., 2013). Increased activation leads to upregulation of *Pvalb*, decreased levels of *Etv1*, and a subsequent shift toward earlier firing at rheobase. We observed *Etv1* expression (data not shown) and corresponding late-firing properties in Pthlh cells, but this did not seem to correlate to *Pvalb* levels, arguing that the type of plasticity seen in the cortex is absent or working through different mechanisms in the striatum. The putative NGCs were indistinguishable between regions. Cortical *Sst*−/*Reln*+ cells, which include *Npy*/*Mia*-expressing Int14, have been shown to rely not on local activity but rather on thalamic inputs for their proper specification and circuit integration (De Marco García et al., 2015). If this is also true in the striatum, it could explain their similarity compared to Pthlh and Sst cells.

Our findings using the PatchSeq protocol, combining electrophysiology with transcriptomics over a large variety of neurons, suggest that these two entities correspond well. We observed significant electrophysiological differences between cortical and striatal *Pvalb*-expressing cells that coincided with significantly different levels of expression of genes known to affect those same electrophysiological properties. This provides support to the finding that molecular information correlates with electrophysiological properties (Földy et al., 2016). However, clustering of neurons into subtypes based on transcriptomic data in an unsupervised manner seems to be more powerful with larger separation between groups.

## STAR★Methods

### Key Resources Table

**Table:** 

REAGENT or RESOURCE	SOURCE	IDENTIFIER
**Antibodies**		

Chicken anti EGFP	Abcam	ab13970; RRID: AB_300798
Rabbit anti CCK	Frontiers institute Co., Ltd	CCK-pro-Rb-Af350; RRID: AB_2571674
Goat anti chicken Alexa 488	Invitrogen	A-11039; RRID: AB_2534096
Goat anti rabbit Alexa 555	Invitrogen	A-21428; RRID: AB_2535849

**Critical Commercial Assays**		

RNAscope Flourescent Multiplex	Advanced Cell Diagnostics biotechne	320850
RNAscope Multiplex Flourescent v2	Advanced Cell Diagnostics biotechne	323110
Papain dissociation system	Worthington	LK003150

**Deposited Data**		

The accession numbers for the scRNAseq data sets reported in this papers is (GEO): Dataset A (GSE97478) and Dataset B (GSE106707)	GEO	GSE106708 (both data sets)

**Experimental Models: Organisms/Strains**		

Mouse: B6;CBA;CD1-Tg(Lhx6-icre)1Kess/J	The Jackson Laboratory	https://www.jax.org/strain/026555
Mouse: Tg(Htr3a-EGFP)DH30Gsat	GENSAT project	http://www.informatics.jax.org/allele/MGI:3846657
Mouse: B6;129P2-Pvalbtm1(cre)Arbr/J	The Jackson Laboratory	https://www.jax.org/strain/008069
Mouse: CD1 wt (RjOrl:SWISS)	Janvier labs	https://www.janvier-labs.com/rodent-research-models-services/research-models/per-species/outbred-mice/product/swiss.html
Mouse: C57BL/6J wt	Janvier labs	https://www.janvier-labs.com/rodent-research-models-services/research-models/per-species/inbred-mice/product/c57bl6jrj.html

**Oligonucleotides**		

Please see Table S6 for complete list of oligonucleotides		

**Software and Algorithms**		

IMARIS software	Bitplane	http://www.bitplane.com
R-studio version 0.99.451	The R Project for Statistical Computing	https://cran.r-project.org/mirrors.html
R version 3.4.2	The R Project for Statistical Computing	https://cran.r-project.org/mirrors.html
MATLAB	MathWorks	https://www.mathworks.com/products/matlab.html
BackSPIN V2 MATLAB	Github	https://github.com/linnarsson-lab/BackSPIN
PatchSeq calling algorithm (EWCE)	Github	https://github.com/NathanSkene/EWCE/
Latent factor analysis code (NBpca)	Github	https://github.com/cortex-lab/Transcriptomics

### Contact for Reagent and Resource Sharing

Further information and requests for resources and reagents should be directed to and will be fulfilled by the Lead Contact: Jens Hjerling-Leffler (jens.hjerling-leffler@ki.se).

### Experimental Model and Subject Details

#### Animals

For the striatal scRNASeq experiments we used transgenic mouse lines *Lhx6*^Cre^ (Fogarty et al., 2007) crossed onto a Rosa26-tdTomato strain, and the BAC transgenic mouse 5HT3a^EGFP^, where EGFP is expressed under the control of the *Htr3a* promoter (GENSAT project, Rockefeller University, NY, USA).

For the single cell experiments in Dataset A mice between p22-p28 (n = 28, 14 females and 14 males) were used and for Dataset B mice from p21-p26 (n = 4, 3 males and 1 female) and p55-p76 (n = 8, 4 females and 4 males). For scRNaseq of cortical Pvalb neurons cells was isolated from *Pvalb-IRES-*cre (Hippenmeyer et al., 2005) crossed onto a tdTomato mice. For electrophysiological recordings and PatchSeq experiments, we used p15-p43 5HT3a^EGFP^ (n = 29, 19 females, 10 males) and *Pvalb*^cre^ (n = 9, 4 females, 5 males) crossed onto a tdTomato or RCE reporter (Sousa et al., 2009). I*n situ* hybridizations: a) for quantification of *Pthlh*, *Pvalb*, *Trh*, *Chodl*, *Tac1*, and *Sst* in Figures 2, 3, and 4 were performed on wild-type CD1 mice, p25, 6 (3 females and 3 males) 4 (2 females and 2 males) or 3 (2 females and 1 male) respectively; b) for validation of *Cck* expressing cells in Figure 2 (CD1 mice, 2 males, p82 and p25), c) for quantification of *Pthlh* and *Pvalb* in older mice (Figure S2) wild-type C57BL/6J (3 males), and d) for quantification of *Pthlh*, *Pvalb* in the Pvalb^cre^::RCE mouse together with EGFP immunohistochemistry in Figure S4 (p28, 3 mice, 2 females and 1 male). Immunohistochemistry of CCK was performed on 5HT3a^EGFP^ mice (p56, n = 2). All other wild-type or transgenic mice were on a mixed CD1 background. Mice were housed on a standard 12+12 light/dark cycle with 2-5 mice per cage, with food and water available *ad libitum*. All tissue was obtained following guidelines and permissions from the local ethics committee, Stockholm Norra Djurförsöksetisks Nämd (N282/14), and Swiss National and Institutional guidelines.

### Method Details

#### Tissue dissociation

Dorsal striatum (bregma, AP: 1.42 to −0.58 mm) was dissociated into a single cell suspension as described previously (Zeisel et al., 2015). Mice were deeply anesthetized with a mixture of ketamine/xylazine (80mg/kg; 10mg/kg), and the brain was quickly dissected and transferred to ice-cold oxygenated cutting solution in mM (87 NaCl, 2.5 KCl, 1.25 NaH2PO4, 26 NaHCO3, 75 sucrose, 20 glucose, 1 CaCl2, and 2 MgSO4) and kept in the same solution during sectioning on a vibratome (VT1200 S, Leica) in 300 μm thick slices. For Dataset B, a cutting solution allowing faster recovery of aged cells was used, containing (in mM) 93 NMDG, 2.5 KCl, 1.2 NaH2PO4, 30 NaHCO3, 20 HEPES, 25 Glucose, 5 sodium ascorbate, 2 thiourea, 3 sodium pyruvate, 10 MgSO4^∗^7H2O, 0.5 CaCl2^∗^2H2O and 12 N-acetyl-L-cysteine. The pH was adjusted to 7.4 using HCl.

From each slice the dorsal striatum was dissected alone (Dataset A) or together with the subventricular zone (Dataset B). The tissue was then dissociated using the Papain dissociation system (Worthington) following the manufacturer’s instructions. All the solutions were oxygenated for at least 10 minutes with a mixture of 5% CO2 in O2 (Labline). Oxygenation and a short time of dissection were crucial to keep a high rate of survival in the cell suspension. After this, the cell suspension obtained was filtered with 20 μm filter (Partec) and kept in cold HBSS solution (SIGMA) with 0.2% BSA and 0.3% glucose. To obtain additional cortical Pvalb cells, S1 cortex from *Pvalb*^cre^::tdTomato mice were gently dissociated in 1 mL ACSF-D solution containing 1.1 mM EDTA, 10 mM L-Cysteine and 15U papain, activated for 15-30 min at 37°C. After dissociation the cell suspension was filtered (30 μm mesh) into 1mL of ACSF-D with 0.5% BSA and damaged cells stained with 0.1% Propidium Iodide (PI).

#### FACS

Dissociated cells were FACS sorted based on fluorescence (RFP+ and EGFP+) either into a tube, prior to running them on the C1-STRT (Dataset A), or directly onto the STRT-seq-2i (Dataset B) platform. For Dataset A, BD FACSAria” III Cell Sorter B5/R3/V3 system was used to collect both fluorescent and non-fluorescent populations from Lhx6^cre^::R26R-tdTomato and 5HT3a^EGFP^ mice, followed by a subsequent manual loading into the C1 chip (Fluidigm system). For the cortico-striatal comparison dataset cortical *Pvalb*^cre^::TdT positive cells were sorted on a FACS ARIA II directly into 3 μL of ice cold ACSF-D with 0.5% BSA in the cell collection chamber of a Fluidigm C1 chip to a final concentration 100-150 cells/μL. The collected cells were processed immediately after FACS on the Fluidigm C1 System according to the C1-STRT protocol. For Dataset B Lhx6^cre^::R26R-tdTomato and 5HT3a^EGFP^ positive cells only were sorted using BD FACSAria II SORP, straight onto the chip array (2400 cells/chip prefilled with 50 nL lysis buffer).

#### Cell capture and imaging for Dataset A

A cell suspension obtained after FACS sorting was concentrated at a range of 600-1000 cells/μL. C1 Suspension Reagent was added (all ‘C1’ reagents were from Fluidigm, Inc.) in a ratio of 4 μL to every 7 μL cell suspension as previously described (Zeisel et al., 2015). 11 μL of the cell suspension mix was loaded on a C1 Single-Cell AutoPrep IFC microfluidic chip designed for 10- to 17-μm cells, and the chip was then processed on a Fluidigm C1 instrument using the ‘mRNA *Seq: Cell Load (1772x/1773x)’* script (30 min at 4°C). The plate was then transferred to an automated microscope (Nikon TE2000E), where a brightfield and RFP or EGFP fluorescence image (20 × magnification) was acquired for each capture site using μManager (http://micro-manager.org/ (2)), which took < 15 min. Quality of cells and control for doublets was performed after each experiment as described in (Zeisel et al., 2015).

#### Lysis, reverse transcription and PCR

C1 chips were processed as described in (Zeisel et al., 2015). Lysis mix (0.15% Triton X-100, 1 U/μL TaKaRa RNase inhibitor, 4 μM Reverse Transcription (RT) primer C1-P1-T31, 5% C1 Loading Reagent and 1:50,000 Life Technologies ERCC Spike-In Mix 1), RT-mix (1 × SuperScript II First-Strand Buffer, 3 mM MgCl2, 1.5 mM dNTP, 4 mM DTT, 3.3% C1 Loading Reagent, 1.8 μM template-switching oligo C1-P1-RNA-TSO, 1.5 U/μL TaKaRa RNase inhibitor and 18 U/μL Life Technologies Superscript II reverse transcriptase) and PCR mix (1.1 × Clontech Advantage2 PCR buffer, 440 μM dNTP, 530 nM PCR primer C1-P1-PCR-2, 5% C1 Loading Reagent and 2 × Advantage2 Polymerase Mix) were added to the chip. After returning to the Fluidigm C1 instrument the ‘*mRNA Seq: RT + Amp (1772x/1773x)*’ script was run, including lysis, RT and 21 cycles of PCR. Thereafter the amplified cDNA wad quantified using an Alignet BioAnalyzer (with an average yield of around 1 ng/ul). All primer sequences can be found in Table S6.

STRT-seq-2i chip arrays were processed as described in (Hochgerner et al., 2017).

Cells were lysed at 72 °C for 3 min in the following solution (500 nM STRT-P1-T31, 4.5 nM dNTP, 2% Triton X-100, 20 mM DTT, 1.5 U/μl TaKaRa RNase Inhibitor). RT mix (2.1X SuperScript II First-Strand Buffer, 12.6 mM MgCl2, 1.79 M betaine, 14.7 U/μl SuperScript II, 1.58 U/μl TaKaRa RNase Inhibitor, 10.5 μM P1B-UMI-RNA-TSO) was then added and the RT was carried out at 42 °C for 90 minutes. Thereafter, the cDNA was amplified using 32 index primers (DI-P1A-idx[1–32]-P1B and PCR (1X KAPA HiFi Ready Mix supplemented with 0.2 mM dNTP, 100 nM DI-PCR-P1A). The PCR was run with the following protocol: 95 °C 3 min. 5 cycles: 98 °C 30 sec, 67 °C 1 min, 72 °C 6 min. 15 cycles: 98 °C 30 sec, 68 °C 30 sec, 72 °C 6 min. 72 °C 5 min, 10 °C hold. All primer sequences can be found in Table S6.

#### Tagmentation and isolation of 5′ fragments

For both C1 and the STRT-seq-2i samples tagmentation of amplified cDNA was performed by fragmentation and incorporation of barcoded adaptors using 96 distinct transposome stocks with unique barcode sequences (10X: 6.25 μM barcoded adaptor C1-TN5-[1-96], 40% glycerol, 6.25 μM Tn5 transposase).

For C1 samples 6 ul cDNA was combined with 2.5 μL of the 10 × transposome stock, 5 μL tagmentation buffer (50 mM TAPS-NaOH, pH 8.5, 25 mM MgCl2 and 50% DMF) and 11.5 μL nuclease-free water. Upon 5 min incubation at 55°C the samples were cooled on ice.

Dynabeads MyOne Streptavidin C1 beads (Invitrogen) were washed and and resuspended (1:20) in BWT (10 mM Tris-HCl, pH 7.5, 1 mM EDTA, 2 M NaCl, 0.02% Tween-20). The beads were then added to the tagmentation reaction (1:20) and incubated at room temperature for 15 min.

All samples were pooled and upon immobilization of the beads and the supernatant was removed. The beads were then resuspended in 100 μL TNT (20 mM Tris, pH 7.5, 50 mM NaCl, 0.02% Tween), washed in 100 μL QIAGEN Qiaquick PB, and again in 100 μL TNT. 100 μL restriction mix (1 × NEB CutSmart, 0.4 U/μL PvuI- HF enzyme) was then added to the reaction, designed to cleave 3′ fragments carrying the PvuI recognition site. The mix was incubated for 1 h at 37°C, then washed (3 x TNT). To elute the cDNA, 30 or 50 ul nuclease-free water was added and incubated for 10 min at 70°C and after beads were bound to magnet the supernatant was collected. To remove short fragments, Ampure beads (Beckman Coulter) were used at 1.8x or 1.5x the total volume of the sample.

The STRT-seq-2i samples were processed in a similar way with some minor changes described below (Hochgerner et al., 2017). The TN5 reactions had the following composition (3ul transposome, 2ul cDNA 1x CutSmart buffer-NEB, total volume 20 ul) and incubated at 55°C for 20 min. BB buffer (10 mM Tris HCl pH 7.5, 5 mM EDTA, 250 mM NaCl, 0.5% SDS) was used for Dynabead dilution. In addition to TNT, remaining adaptors were cleaned by adding 10 μl ExoSAP IT (Affymetrix) and incubating 15 min 37 °C. Also a second PCR was run using the following mix (1X KAPA HiFi Ready Mix, 200 mM 4K-P1_2ndPCR, 200 nM P2_4K_2ndPCR), and cycled (95 °C 2 min. 8 cycles: 98 °C 30 sec, 65 °C 10 sec, 72 °C 20 sec. 72 °C 5 min, 10 °C hold). The supernatant was collected, cleaned with 0.7X volumes of AmPure beads and eluted. The eluate was bound to 0.5X volumes of AmPure beads, the supernatant transferred, again bound in 1X volume of AmPure beads, and finally eluted in EB. All primer sequences can be found in Table S6.

#### Illumina high-throughput sequencing and molecule counts

C1 library quality and concentration was quantified using qPCR and KAPA Library Quant (Kapa Biosystems). Library fragment length was estimated using Bioanalyzer of a reamplified (12 cycles) library.

Sequencing was performed on an Illumina HiSeq 2000 instrument using C1-P1-PCR-2 as the read 1 primer, and C1- TN5-U as the index read primer (see Key Resources Table). Reads of 50 bp were generated along with 8 bp index reads corresponding to the cell-specific barcode. Each read was expected to start with a 6 bp unique molecular identifier (UMI), followed by 3-5 guanines, followed by the 5′ end of the transcript

STRT-seq-2i library quality control and quantification was performed using Bioanalyzer. Sequencing was performed on an Illumina HiSeq 2000 or 2500 instrument and the single-End 50 cycle kit using the Read1 DI-Read1-Seq, Index 1 STRT-Tn5-U and Index 2 DI-idxP1A-Seq (see Key Resources Table). Reads of 45 bp were generated, starting with a 6 bp UMI, followed by 3 guanidines, and the 5′ transcript. The two index reads of 8 and 5 bp represents Index 1 (subarray barcode) and Index 2 (well barcode), respectively.

#### Histology

##### *In situ* hybridization

Brains from wild-type CD1 mice were dissected and directly embedded in OCT cryomount (Histolab Products AB), frozen on dry ice and kept at −80°C. Coronal brain sections (10 μm thickness; bregma, AP: 1.42 to −0.58 mm) were obtained using a cryostat (Leica Biosystems) and collected in 22 series. *In situ* hybridization using the RNAscope technology (Advanced Cell Diagnostics biotechne) was performed for the following genes: *Chodl*, *Sst*, *Pthlh*, *Trh*, *Pvalb, Tac1, Mia*, *Npy* and *Cck* according to the manufacture’s instructions.

##### Immunofluorescence

Pvalb^cre^::RCE or 5HT3a^EGFP^ mice were perfused with PBS followed by paraformaldehyde 4% and brains were dissected and postfixated during 3 hours, cryoprotected in sucrose solution 30% in PBS, embedded in OCT, and kept at −80°C.

After that, sections of 14 μm (Pvalb^cre^::RCE mice) or 50 μm (5HT3a^EGFP^ mice) thickness were obtained using the cryostat described above. In the Pvalb^cre^::RCE mice the *in situ* hybridization for *Pvalb* and *Pthlh* was performed first according to manufacter’s instructions (Advanced Cell Diagnostics biotechne). Then, immunostaining following the same procedure that described in (Muñoz-Manchado et al., 2016). Antibodies: chicken anti EGFP (Abcam), rabbit anti CCK (Frontiers institute Co., Ltd), goat anti chicken Alexa 488 and goat anti rabbit Alexa 555 (Invitrogen).

#### Image acquisition and analysis

Confocal images were acquired using a Zeiss LSM 700 or a Zeiss LSM 800 microscope. Analysis of *in situ* hybridization images was performed on 2 tile scan confocal images that cover a full hemistriatum (right and left striatum per mouse):

• for the visualization/quantification of *Pthlh*/*Pvalb*/*Trh* (p25, n = 6, 3 males and 3 females; 5 months, n = 3) and *Chodl/Sst* (p25, n = 3) positive cells were manually counted.
• for the visualization/quantification of level of expression *Pthlh/Pvalb* (n = 3), *Th/Trh* (n = 4), *Chodl/Sst/Tac1* (n = 3) *and Sst/Mia/Npy* (n = 3) mRNA molecules per cell were counted using IMARIS software (Bitplane).
• for the visualization/quantification of expression of *Pthlh*, *Pvalb* and EGFP in the Pvalb^Cre^::RCE mouse (n = 3, p28, 844 cells) intensity of fluorescence was measured using IMARIS software (Bitplane). Threshold was set up for each picture with background measure for each channel.

#### Electrophysiological recordings

Whole-cell patch-clamp recordings were performed as previously described (Muñoz-Manchado et al., 2016) on 5HT3a^EGFP^, *Pvalb*^cre^::RCE and *Pvalb*^cre^::tdTomato animals. Prior to brain dissection, animals were transcardially perfused using ice-cold oxygenated cutting solution (in mM): 87 NaCl, 2.5 KCl, 1.25 NaH_2_PO_4_, 26 NaHCO3, 75 sucrose, 12.5 glucose. Patch electrodes (borosilicate glass; resistance 3–8 MΩ; Hilgenberg, GmbH) were filled with 1-2 ul RNase free internal solution containing (in mM): 130 potassium gluconate, 6 NaCl, 10 HEPES, 0.5 EGTA, 4 Na_2_-ATP, 0.35 Na_2_-GTP, 8 Na_2_-phosphocreatine, and 1 U/μl recombinant RNase inhibitor (Takara).

#### Measurement of intrinsic properties

Electrophysiological parameters were acquired using depolarizing and hyperpolarizing current steps as previously described (Muñoz-Manchado et al., 2016)

Resting membrane potential (RMP) and input resistance (Rin) was measured momentarily after membrane rupture. First AP discharged upon 1pA steps of increasingly depolarizing current injections was used to measure following parameters: voltage AP threshold, AP current threshold, AP amplitude, AP half-width, after hyperpolarization (AHP) latency, AHP amplitude. In the case the cells exhibited a biphasic AHP, the difference in amplitude and latency the two phases of the AHP were measured. The cell was further depolarized until firing failure in order to acquire maximum firing frequency, steady frequency, adaptation and inter-event interval (IEI). By using hyperpolarizing steps, H-current-mediated sag was measured as the voltage difference ratio between the peak hyperpolarization and the steady-state response. Also rebound was measured as the amplitude difference from steady state response of hyperpolarizing current. Time constant (τm) was extracted by using an exponential fit to the decay phase of a voltage response to a hyperpolarizing current step (Figure S5).

#### PatchSeq

Cell harvesting and RNA extraction, transcription and PCR amplification

After the recording, weak negative pressure was applied in order to aspirate the recorded cells into the glass capillary patch electrode. Using positive pressure the pipette content was quickly ejected into a 1 μL drop of RNase-free lysis buffer (1 mM C1-P1-T31, 10uM dNTP, 10% Triton X, 100mM DTT, 2U/ul Takara Rnase inhibitor) placed on the side of a 0.2 mL RNase free tight-lock tube (TubeOne). The sample was rapidly spun down (5-10 s) and stored at −80 before reverse transcription (RT). Reverse transcription, PCR amplification and sequencing were manually performed in the same way as for Dataset A as also described in (Fuzik et al., 2016).

### Quantification and Statistical Analysis

#### Clustering analysis (Dataset A)

Single-cell RNaseq data was loaded as unique molecular identifier (UMI) counts in a genes by cells matrix. First, we selected cells with more than 1500 molecules. Before clustering, genes with less than 25 molecules over the whole dataset were removed. We then used BackSPINv2 with the following parameters: splitlev = 6; Nfeture1 = 1000; Nfeture = 200; N_to_backspin = 50; N_to_cells = 500; mean_tresh = 0.05; fdr_th = 0.3; min_gr_cells = 5; min_gr_genes = 3. This resulted in 59 cluster which we then merged into main general categories, astrocytes, cycling, oligodendrocytes, VLMC, endothelial, microglia, vascular smooth muscle, SPN (Gpr88 positive), interneurons. In addition out of the 1412 cells we removed 278 cells either because of doublet suspicious or low quality. 145 were suspected doublets (based on mixed expression profiles), 91 were low quality SPNs and 42 where low quality small clusters. Low quality is indicated by lower general expression and high levels of mitochondrial markers. For further analysis we focused on the neurons. For interneurons clustering, we first roughly selected genes that are more specific to interneurons by simply performing t tests between all interneurons and other cells and including genes with FDR < 5%. To prevent any effect of oligodendrocytes specific genes contamination we used the same procedure and excluded genes that are specific to the oligodendrocytes. In addition, we excluded genes from the activity lists (see above). Next, we ran BackSPINv2 with the parameters: splitlev = 4; Nfeture1 = 500; Nfeture = 300; N_to_backspin = 50; N_to_cells = 200; mean_tresh = 0.05; fdr_th = 0.3; min_gr_cells = 10; min_gr_genes = 5. This resulted in 16 clusters, which were merged into 6 clusters, and an additional 38 cells were removed. A similar procedure was performed to cluster the SPN cells, which we merged into 2 clusters. One SPN cluster (86 cells) was removed due to low quality signal.

tSNE visualization: Figure 1B perplexity = 40, number of genes 300, 1000 iterations, distance as correlation. In Figure 5A perplexity = 40, number of genes 412, initial PCA dimensions = 40, 1000 iterations distance as correlation.

#### Clustering and gradient analysis (Dataset B)

Single-cell UMI counts data from four chip arrays were assembled as Dataset B. We first removed cells with less than 800 UMIs or if the ratio of total UMI / total genes was lower than 1.2. We also removed all cells that are suspected to be non-neuronal which we identify as expressing > 0 molecules of any of the non-neuronal markers *Mog*, *Mbp*, *Aqp4*, *Gja1*, *C1qc*, *Aif1*, *Fn1*, *Cldn5*. Next, data was normalized to have 2000 UMI counts per cell followed by rounding the numbers to the closest integer. We selected 1000 genes using log(CV) versus log(mean) procedure as described before(Zeisel et al., 2015). We then used BackSPINv2 with following parameters: splitlev = 5; Nfeture1 = 500; Nfeture = 100; N_to_backspin = 10; N_to_cells = 500; mean_tresh = 0.01; fdr_th = 0.3; min_gr_cells = 5; min_gr_genes = 10. This resulted in eleven clusters, which were merged into nine after manual inspection and trashing 448 (out of 3865) cells which either showed low quality or were outliers in the tSNE projection. tSNE visualization: perplexity = 20, number of genes 270, 2000 iterations, and correlation as distance. To analyze genes with gradient expression pattern within each cluster (Table S1) we performed the following analysis: For each larger cluster (Pthlh, Npy/Sst and Th) we selected genes which expressed (> 0) in more than 10% of the cells of the cluster and less abundant than 70% in the whole dataset. We then calculated the correlation (Pearson) between the expression of each gene and the tSNE coordinated (both x and y). This analysis was performed independently for each cluster.

#### Robustness of clusters

To assess the robustness of the clusters, we trained a random forest classifier to recognize cluster labels, and then assessed its performance on held-out data (80% training set, 20% test set) using Dataset B. We calculated the average precision and recall, and computed the probability for each cluster that its cells would be classified as another cell type.

#### Latent Factor analysis

Latent factors were calculated for the three largest cell types (Npy/Sst, Pthlh and Th) using Dataset B. To compensate for any batch effects between samples, a column was added to the F0 argument for all but one sample plate, containing a one if the cell came from that plate and a zero otherwise. Genes were included in the latent factor analysis only if they had over three reads in over eight cells (this left 238 genes for Pthlh, 160 for Th and 76 for Npy/Sst). Expression of each cell was normalized so they had the same total expression level. Association between the latent factor and Pvalb/Tac1/Trh expression was evaluated using a negative binomial generalized linear model. Association with patchseq electrophysiology profiles was calculated by first taking the log of patchseq expression values, then multiplying with the latent factor gene weights. Significance of association with the electrophysiology data was evaluated using a linear model.

#### Comparison between striatal and cortical cells

Molecule counts data from interneurons from (Zeisel et al., 2015) with additional cortical *Pvalb*-expressing cells obtained from S1 of *Pvalb*^cre^::tdTomato mice, were merged into one dataset with the striatum interneurons cells. In this previous study (Zeisel et al., 2015) we analyzed both cortical (S1) and hippocampal (CA1) interneurons and found that they cluster into cell types regardless of tissue of origin (i.e., there are larger differences between cell types than across tissues). Because of this, the classification we use as a comparison in this paper is based on both tissues. For tSNE visualization, we selected genes that are enriched for each of the cluster (412 genes) and ran tSNE as described above for Dataset A. For hierarchical clustering of the groups we calculated the average expression of each group after log2(x+1) transformation and normalizing the total number of molecules to 10,000 for each cell. The same set of genes was used when calculating the tSNE projection and the dendrogram. The dendrogram was generated using linkage clustering method (Ward in MATLAB) with correlation as distance.

#### Group pairwise **comparison, correlation and volcano** plots

Group pairwise comparison: we calculated the p value using the Ranksum test (MATLAB ranksum) at the single-cell level. We selected a threshold for significant differentially express q-value < 0.05 and fold change > 2 (both directions).

Multiple correlations: we identified genes that were correlated and anti-correlated with *Pvalb* using Spearman’s rank correlation coefficient (rho). The threshold for significance was set to p-adjust < 0.05 and rho > 0.15.

p values were adjusted to q-values which have the meaning of which FDR level (Benjamini- Hochberg procedure) needed to find the hypothesis significant.

#### Selection of cluster enriched genes and markers

To select markers enriched genes we used the following approach. For each gene i and cluster j calculate the ratios: where E_(i,k) is the expression of gene i in cell k. Those quantities represent the molecule enrichment in the cluster and the fraction of cells express the gene in that cluster respectively. We then combine the two scores while varying the weight given to the fraction of positive cells (see formula below). Rank the gene for every cluster by the score:

$$\begin{array}{l} \mathrm{enric}h_{\mathrm{i,j}}=\frac{1}{k\in j}\sum_{k\in j}E_{\mathrm{i,k}}/\frac{1}{N}\sum_{k}E_{\mathrm{i,k}}\\ \mathrm{posfra}c_{\mathrm{i,j}}=\frac{1}{k\in j}\sum_{k\in j}I\left(E_{\mathrm{i,k}}>0\right)\\ S_{\mathrm{i,j}}=\mathrm{enric}h_{\mathrm{i,j}}\times \mathrm{posfra}c_{\mathrm{i,j}}^{\mathrm{power}} \end{array}$$

where “power” sets the weight for the fraction of positive cell in the cluster. We used power = 0,0.5,1 to rank the genes in every cluster and then use the top X genes as most enriched.

#### PatchSeq analysis

Only sequenced cells with > 2000 mRNA molecules or > 1000 distinct genes detected (excluding mitochondrial and rRNA) were used for the analysis. In addition, neurons with electrophysiological recordings showing an AP half-width larger than 2ms or an AP amplitude lower than 40 mV were excluded. PatchSeq cells were assigned to clusters based on the expression of the 198 highest informative marker genes for the different interneuron populations acquired from BackSPINv2 in Dataset B (heatmap in Figure 1C). The mapping, which is based on bootstrap analysis, allows calculation of the specificity of each gene to each of the cell type using the EWCE package in R (https://github.com/NathanSkene/EWCE). Dataset B was used as a template, including expression profiles of the marker genes in all interneuron populations. To avoid uninformative genes for the PatchSeq dataset to influence the outcome, PatchSeq expression data was then filtered to exclude genes with low variance across the dataset (SD < 1). Bootstrapping approach was then used to assign each of the PatchSeq cells to a cell type. For each gene retained from the PatchSeq data, we multiplied its read count by its specificity metric (based on Dataset B). The bootstrapping was repeated 10,000 times, each time selecting random gene-lists of equal size.

The summed specificity metrics from the bootstrapping resulted in the probability of each PatchSeq cell corresponding to each interneuron population in Dataset B. Only cells with p < 0.05 were assigned to the corresponding cluster, the remaining cells were called “undefined” (28 cells).

#### Statistics

##### Intrinsic properties

Electrophysiological similarities of sequenced cells and non-sequenced cells were visualized using Principal component analysis (PCA) and Hierarchical clustering on the basis of Euclidean distance, on normalized (log transformed) data. Multiple testing was done using unpaired two-sided t tests with Benjamini-Hochberg correction.

##### Comparison of ion channels genes

Comparison of ion channel expression was done using unpaired two-sided t tests with Benjamini-Hochberg correction upon normalization of the data (log transforming).

##### Correlation of gene expression with electrophysiological properties

Pearson’s correlation coefficient was used to measure correlation between electrophysiological parameters and gene expression with p < 0.05 as threshold for significance.

##### Histology

Pearson’s correlation coefficient was used to measure correlation between gene expression or gene expression and location within tissue.

### Data and Software Availability

All statistical tests were performed using R-studio Version 0.99.451 and R version 3.4.2.

#### Data availability

The accession number for the raw data reported in this paper is GEO: GSE106708 (https://www.ncbi.nlm.nih.gov/geo/query/acc.cgi?token=ktylseqizzotrap&acc=GSE106708).

GSE106708 is a Super series containing both Dataset A (GSE97478) and Dataset B (GSE106707) and has this reviewer access token: upyhwumypnoxxsl.

#### Code availability

BackSPIN V2 for MATLAB is available upon requests without restriction

PatchSeq calling algorithm can be found at https://rdrr.io/github/NathanSkene/EWCE/

Latent factor analysis code is available from https://github.com/cortex-lab/Transcriptomics (the function is called NBpca).

## Supplemental Information

Document S1. Supplemental Figures S1–S7 and Tables S3 and S5

Table S1. Genes Weights in the Latent Factor Analysis of Dataset B for the Three Largest Interneuron Clusters: Pthlh, Npy+Sst+, Th, Related to Figure 3

Table S2. Genes Correlated with Pvalb Expression in the Pthlh Population (Dataset B, Spearman’s Rank Correlation Coefficient), Related to Figure 4

Table S4. Results of Pairwise Comparison of Pvalb-Expressing Cells from Cortex and Striatum Using Wilcoxon Rank Sum Test, Related to Figure 6

Table S6. Oligos Used for Tagmentation of cDNA before Sequencing, Related to the Key Resources Table and Figure 1

Document S2. Article plus Supplemental Information
