# Supplementary material for: Diversity of Interneurons in the Dorsal Striatum Revealed by Single-Cell RNA Sequencing and PatchSeq
Source: Cell Rep. 2018 Aug 24;24(8):2179–2190.e7. doi: 10.1016/j.celrep.2018.07.053 (PMC6117871; doi:10.1016/j.celrep.2018.07.053)
Supplement: Document S1. Supplemental Figures S1–S7 and Tables S3 and S5 [file mmc1.pdf]

Cell Reports, Volume *24*

Supplemental Information

Diversity of Interneurons in the Dorsal Striatum Revealed by Single-Cell RNA Sequencing and PatchSeq

Ana B. Muñoz-Manchado, Carolina Bengtsson Gonzales, Amit Zeisel, Hermany Munguba, Bo Bekkouche, Nathan G. Skene, Peter Lönnerberg, Jesper Ryge, Kenneth D. Harris, Sten Linnarsson, and Jens Hjerling-Leffler

Figure S1

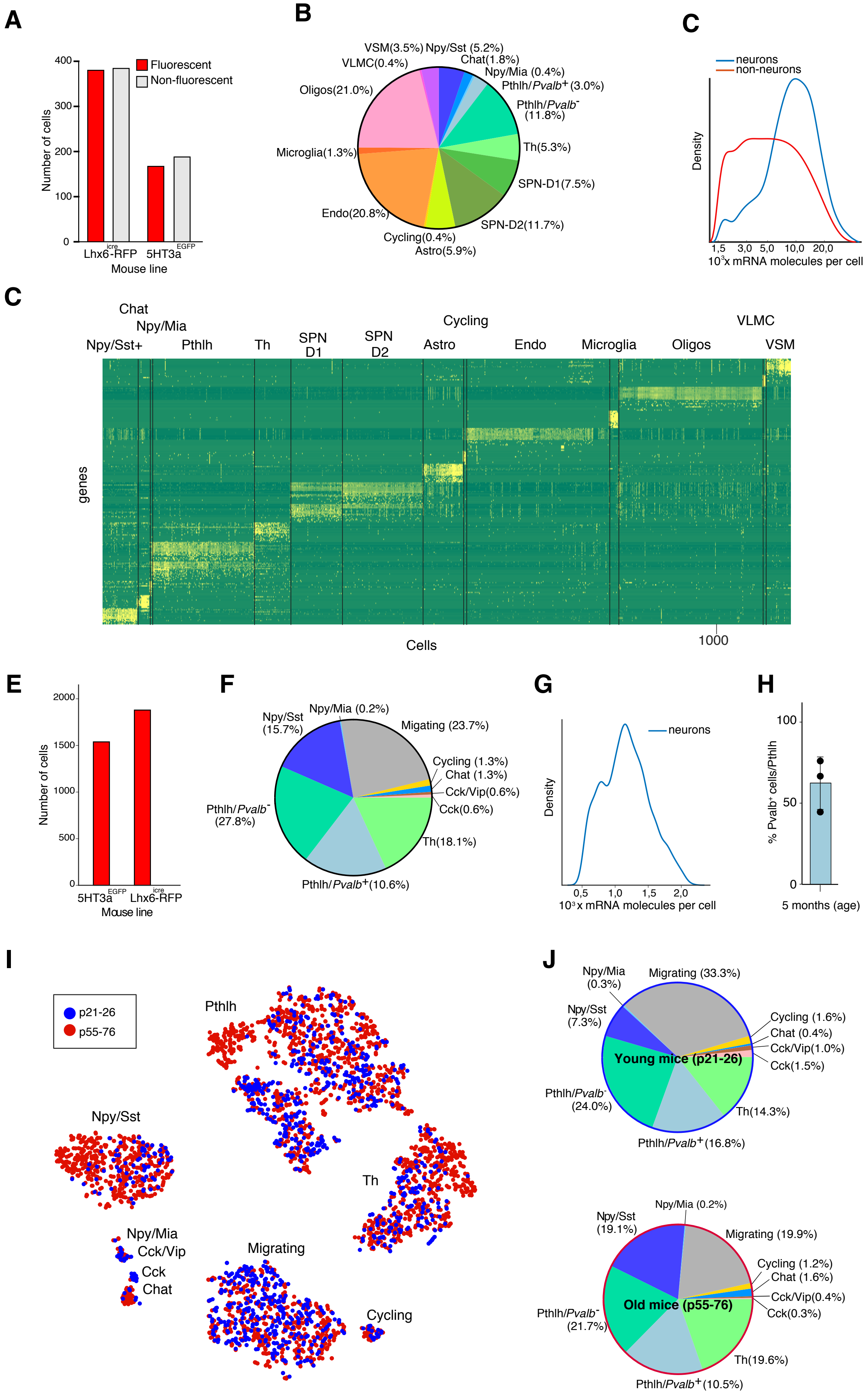

A
Fluorescent
Non-fluorescent
Number of cells
Lhx6icre-RFP
5HT3aEGFP
Mouse line
B
VSM(3.5%) Npy/Sst (5.2%)
VLMC(0.4%)
Chat(1.8%)
Npy/Mia (0.4%)
Pthlh/Pvalb+ (3.0%)
Oligos(21.0%)
Pthlh/Pvalb- (11.8%)
Microglia(1.3%)
Th(5.3%)
SPN-D1(7.5%)
Endo(20.8%)
SPN-D2(11.7%)
Cycling(0.4%)
Astro(5.9%)
C
neurons
non-neurons
Density
10³x mRNA molecules per cell
C
Chat
Npy/Mia
Npy/Sst+
Pthlh
Th
SPN D1
SPN D2
Cycling
Astro
Endo
Microglia
Oligos
VLMC
VSM
genes
Cells
1000
E
Number of cells
5HT3aEGFP
Lhx6icre-RFP
Mouse line
F
Npy/Sst (15.7%)
Npy/Mia (0.2%)
Migating (23.7%)
Cycling (1.3%)
Chat (1.3%)
Cck/Vip(0.6%)
Cck(0.6%)
Pthlh/Pvalb- (27.8%)
Th(18.1%)
Pthlh/Pvalb+(10.6%)
G
neurons
Density
10³ x mRNA molecules per cell
H
% Pvalb+ cells/Pthlh
5 months (age)
I
p21-26
p55-76
Pthlh
Npy/Sst
Th
Npy/Mia
Cck/Vip
Cck
Chat
Migrating
Cycling
J
Npy/Mia (0.3%)
Migrating (33.3%)
Npy/Sst (7.3%)
Cycling (1.6%)
Chat (0.4%)
Cck/Vip(1.0%)
Young mice (p21-26)
Cck(1.5%)
Pthlh/Pvalb- (24.0%)
Th(14.3%)
Pthlh/Pvalb+(16.8%)
Npy/Mia (0.2%)
Npy/Sst (19.1%)
Migrating (19.9%)
Cycling (1.2%)
Chat (1.6%)
Cck/Vip(0.4%)
Old mice (p55-76)
Pthlh/Pvalb- (21.7%)
Cck(0.3%)
Th(19.6%)
Pthlh/Pvalb+(10.5%)

Figure S1. Cell populations in the dorsal striatum (Dataset A and B) and how they are affected by age. Related to Figure 1.

(A) Bar plots showing the number of fluorescent and non-fluorescent cells obtained in Dataset A upon FACS sorting of dorsal striatum from Lhx6cre::R26R-tdTomato and 5HT3aEGFP mouse lines including: 21% oligodendrocytes (Oligos; expressing *Mog, Mag* and *Plp1*), 5.9% astrocytes (Astro; expressing *Aldoc, Gja1* and *Slc1a2*), 3.5% vascular smooth muscle cells (VSM; expressing *Mylk, Acta2* and *Tpm2*), 20.8% endothelial cells (Endo; *Sparc, Ly6c1* and *Car4*), 0.4% cycling cells (Cycling; *Cdk1* and *Prc1*), 0.4% vascular and leptomeningeal cells (VLMC; *Pdgfra* and *Igf2*) and 1.2% microglia (*Cx3cr1* and *Fcrls*).

(B) Pie chart showing the percentages of the different cellular populations obtained after analysis of the 1135 sequenced cells in Dataset A.

(C) Neuronal and non-neuronal cells at different mRNA expression levels from Dataset A.

(D) Heatmap of same populations shown in (B), clustered using BackSPINv2.

(E) Bar plots showing the number of fluorescent cells obtained in Dataset B.

(F) Pie chart showing the percentages of the different cellular populations obtained after analysis of the 3417 sequenced cells in Dataset B.

(G) Density of neuronal cells at different mRNA expression levels from Dataset B.

(H), Same tSNE as in D (Dataset B) with the cells colored according to mouse age, young cells (p21-26) in blue and older cells (p55-76) in red.

(I) Percentages of *Pthlh* positive cells that co-express *Pvalb*, in old mice (5 months). Error bars represent mean ± SEM.

(J) Pie chart showing the percentages of the different cellular populations in the same age groups as in (A) obtained after BackSPINv2 analysis.

Figure S2

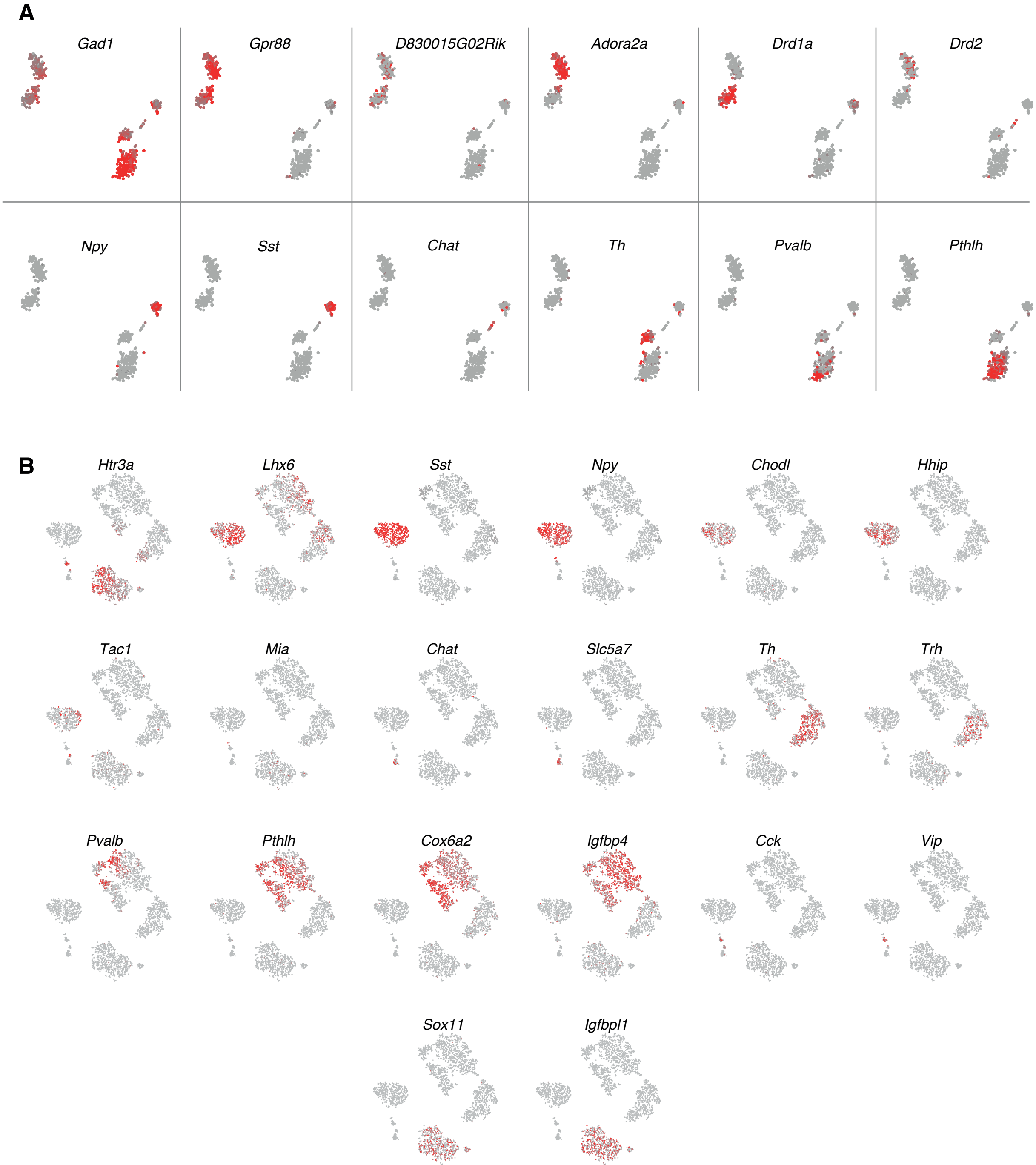

Figure S2. Markers expressions in Dataset A and B. Related to Figures 1 and 2. (A) Expression of known and suggested markers (high expression in red) of the neuronal populations obtained in Dataset A, visualized in the same tSNE plot as in Figure 1B. (B) Same as in (A) but for Dataset B, visualized in the same tSNE plot as in Figure 1D.

Figure S3

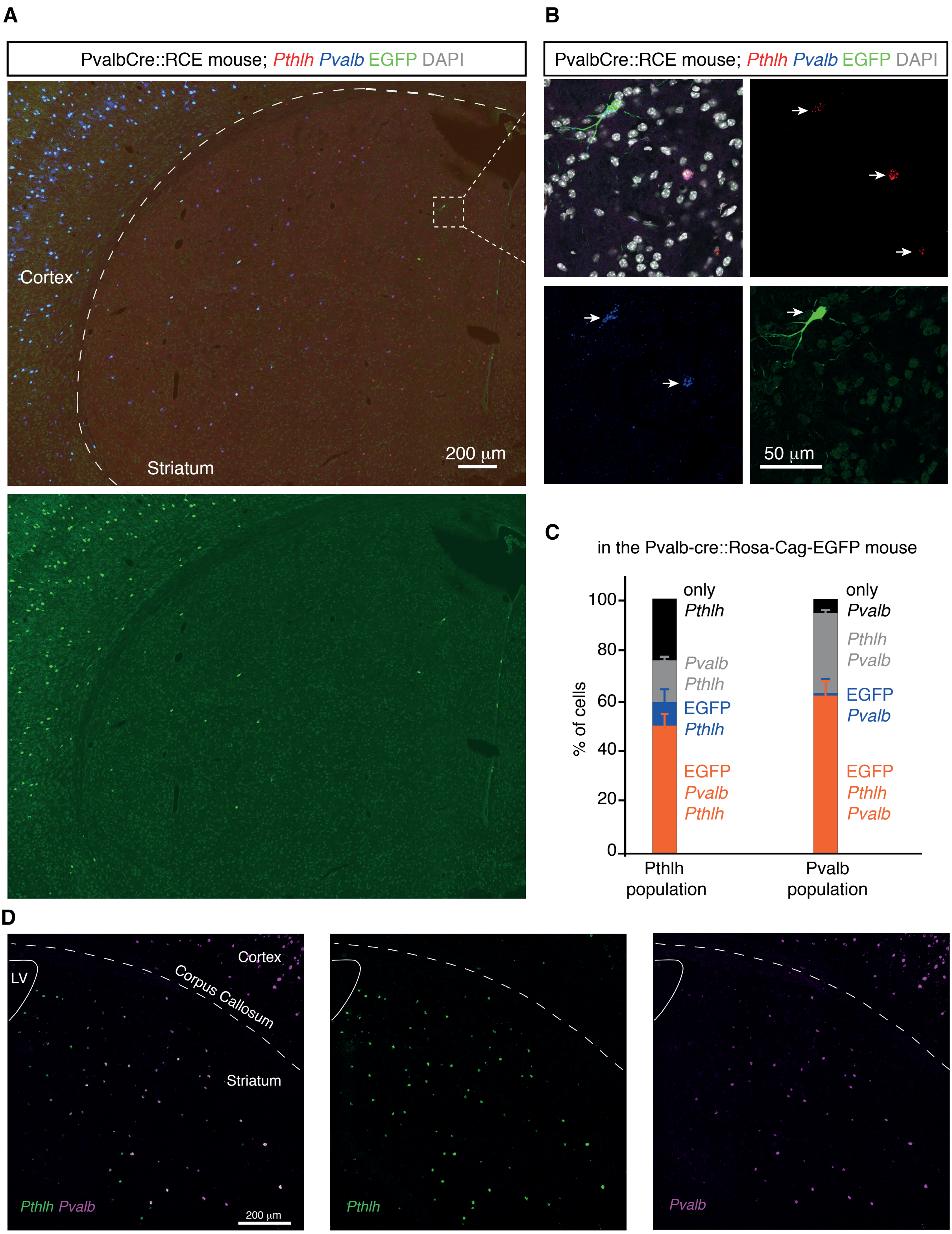

Figure S3. Pthlh and Pvalb populations in the PvalbCre::RCE and in wild-type mice. Related to Figures 4, 5 and 7.

(A) Representative in situ hybridization for *Pthlh* and *Pvalb* (in red and blue respectively) together with immunostaining for EGFP (in green) in the dorsal striatum showing the labeling of *Pthlh*+ and *Pvalb*+ cells in the mouse line PvalbCre::RCE mouse line.

(B) Higher magnification from (A).

(C) Quantification of the staining described in (A), in percentage of cells with overlapping expression for Pthlh and/or Pvalb and/or EGFP expression in *Pthlh*+ and *Pvalb*+ cells respectively (3 mice, p28, 844 cells).

(D) Individual channels for the in situ hybridization shown in Figure 4C. *Pthlh* in green and *Pvalb* in magenta.

Figure S4

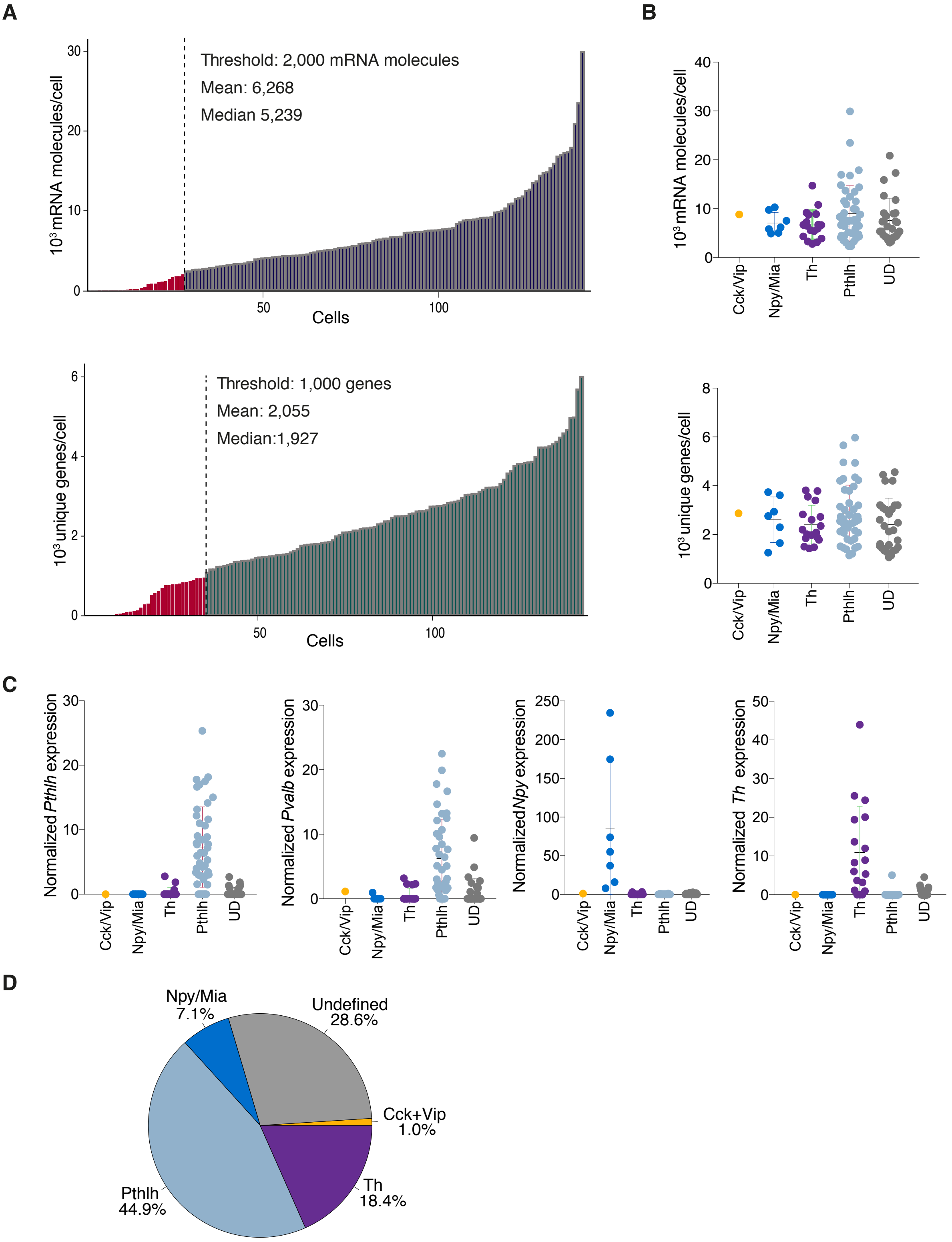

Figure S4. PatchSeq quality control. Related to Figure 5.
(A) Barplot showing molecular count and gene count of each sequenced cell after PatchSeq approach. Cells with a gene count of less than 1000 or molecule count less than 2000 were excluded from the analysis.
(B) Dotplots of total mRNA molecules and unique gene counts of each cell in the different cell classes obtained after mapping them onto BackSPINv2 clustering of Dataset B.
(C) Dotplots of normalized marker expression (Th, Npy, Pthlh and Pvalb) in cells within the different classes.
(D) Pie chart showing the percentages of the different cellular populations in the same groups. Cells that were assigned with a p>0.05 are named undefined. Error bars represent mean ± SEM.

Figure S5

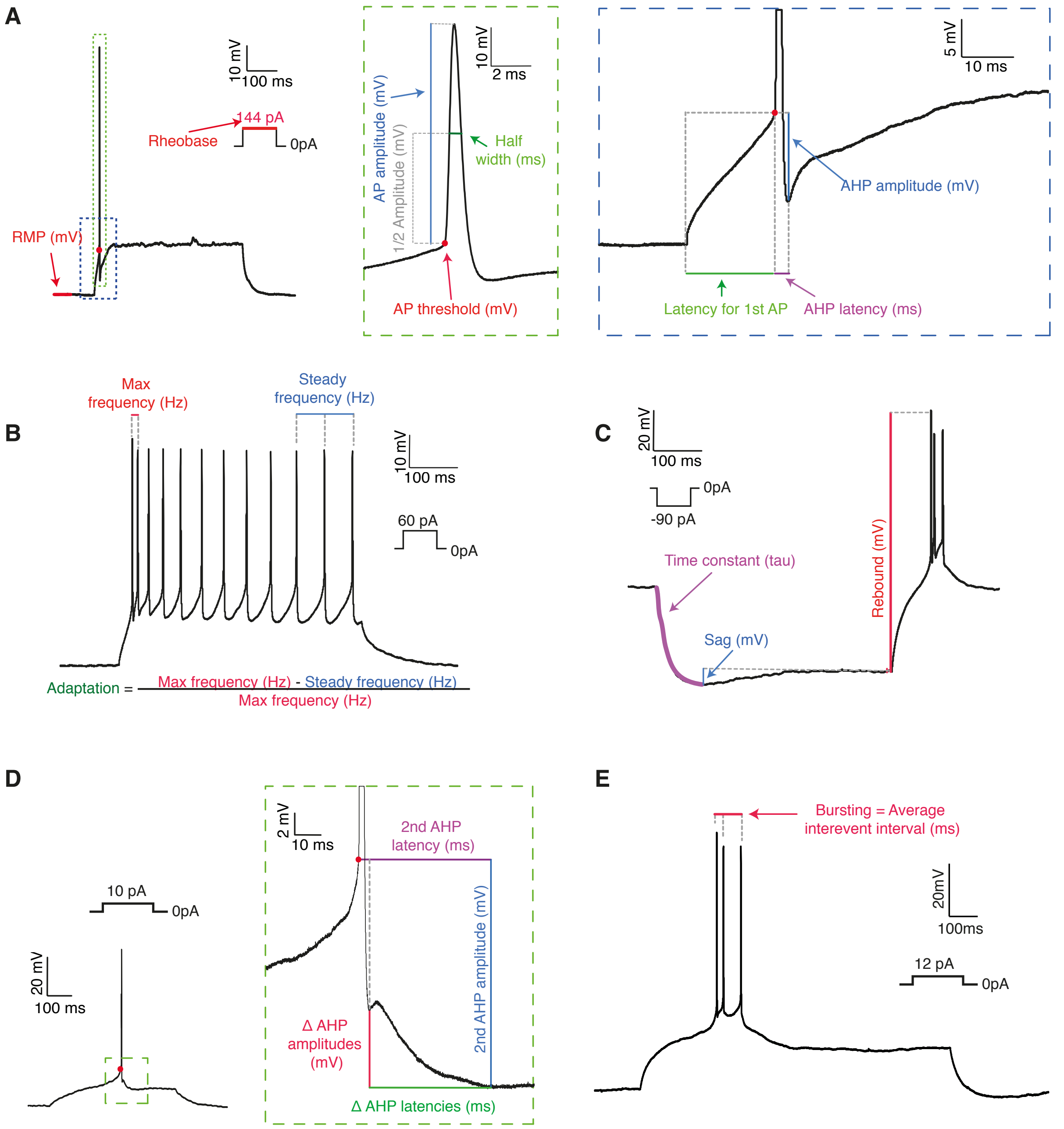

Figure S5. Electrophysiological parameters measured. Related to Figures 5 and 7.

(A) Schematic showing how Resting membrane potential (RMP), Rheobase, Action Potential (AP) amplitude, AP half width, AP threshold, Latency for first AP, After-hyperpolarization (AHP) latency and AHP amplitude were measured.

(B) Illustration showing how Max frequency, Steady frequency and adaptation were measured.

(C) Schematic showing how Time constant, Sag ratio and Rebound were measured.

(D) Representation of how 2nd AHP latency, 2nd AHP amplitude and the difference to the 1st AHP were measured in cells with biphasic AHPs.

(E) Illustration of how bursting was detected using average Interevent interval (IEI) of first trace with 2-3 AP after rheobase.

Figure S6

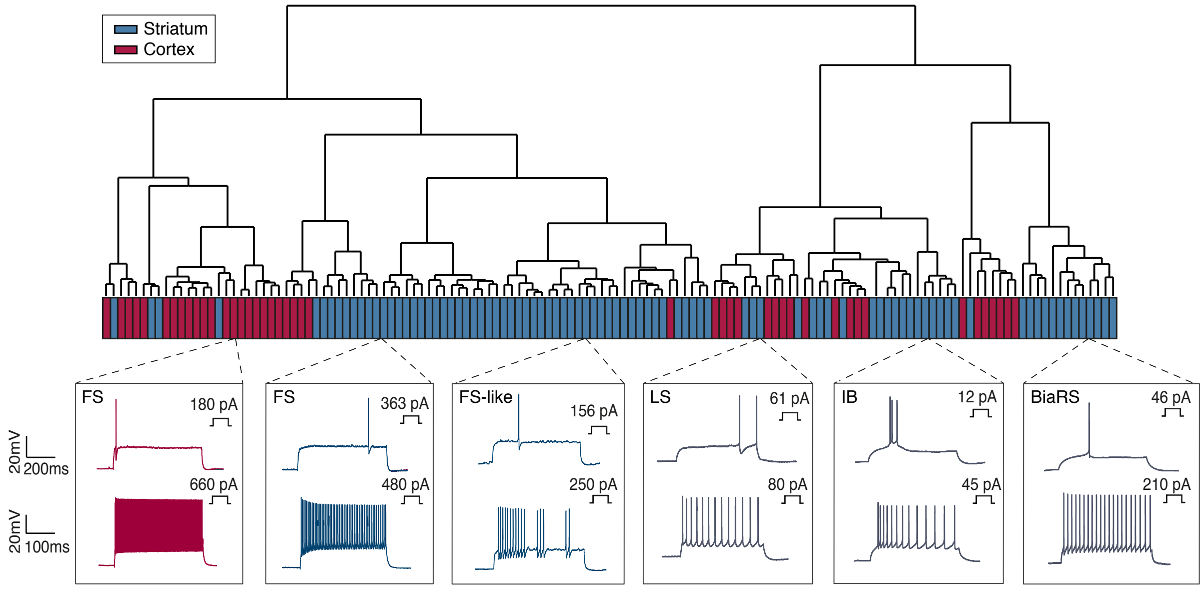

Figure S6. Clustering dendrogram of electrophysiological properties from Pvalbcre+ and 5HT3aEGFP+ cells from striatum and cortex. Related to Figure 7.
(A) Hierarchical clustering based on Euclidean distance of the same cells analyzed in Fig 7A-B, with representative traces below. Acronyms: FS, fast-spiking; FS-like: fast-spiking-like; IB, Intrinsic bursting; LS, late-spiking; BiaRS, bi-phasic AHP regular spiking.

Figure S7

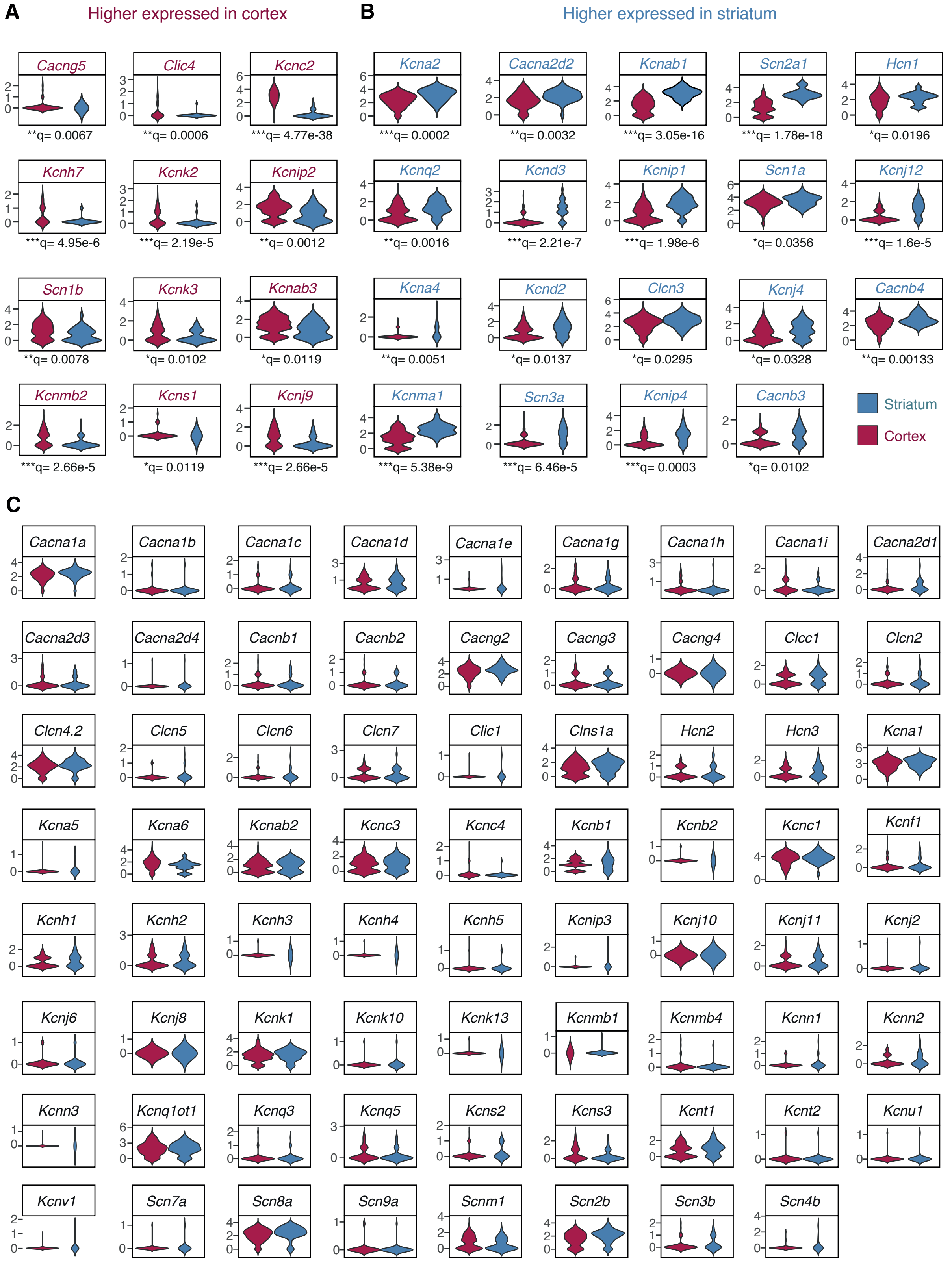

Figure S7. Differentially expressed ion channels in cortical and striatal Pvalb expressing cells. Related to Figure 7.

Differentially expressed ion channels in cells from Dataset A (striatum; 34) together with additional S1 cortical cells (122) processed with the same protocol, were tested upon log transformation using unpaired t-test with Benjamini-Hochberg correction for multiple testing.

(A) Violin plots showing ion channels significantly higher expressed in cortex (red) than striatum (blue) Pvalb+ ($q < 0.05$).

(B) Same than a but showing the ones significantly higher expressed in striatum.

(C) Violin plots of ion channels that did not differ in expression between the structures ($q > 0.05$).

	Pthlh (44 cells)		Th (18 cells)		Npy/Mia (7 cells)		Cck/Vip (1 cell)	
	**Mean**	**SD**	**Mean**	**SD**	**Mean**	**SD**	**Mean**	**SD**
**Input resistance (MΩ)**	144.70	78.14	307.78	157.46	195.14	102.32	170.00	0.00
**RMP (mV)**	-69.07	5.84	-62.00	7.77	-64.00	6.83	-73.00	0.00
**Time constant (τ)**	8.97	13.05	28.14	16.22	13.35	4.55	3.23	0.00
**Rheobase (pA)**	165.57	95.55	29.32	35.63	63.43	42.79	198.00	0.00
**AP treshold (mV)**	-37.05	3.69	-38.36	3.55	-33.56	3.72	-34.24	0.00
**AP amplitude (mV)**	62.11	8.28	66.45	8.63	67.71	8.90	40.10	0.00
**Half width (ms)**	0.82	0.25	1.09	0.47	1.63	0.53	0.73	0.00
**AHP amplitude (mV)**	16.07	3.30	11.14	3.36	21.11	2.21	10.35	0.00
**AHP latency (ms)**	5.86	4.54	17.42	37.67	22.74	9.31	9.42	0.00
**Latency for first AP (ms)**	413.88	235.35	384.69	224.79	524.40	256.70	333.80	0.00
**AP repolarization latency (s)**	0.07	0.05	0.09	0.08	0.21	0.10	0.05	0.00
**Amplitude difference biphasic AHP (mV)**	-0.05	0.32	1.47	2.16	0.00	0.00	0.00	0.00
**Latency difference biphasic AHP (ms)**	0.00	0.00	0.05	0.09	0.00	0.00	0.00	0.00
**Sag ratio**	-0.01	0.01	-0.03	0.01	-0.02	0.01	0.02	0.00
**Max frequency (Hz)**	118.93	68.79	97.40	49.37	51.52	17.73	111.98	0.00
**Steady frequency (Hz)**	61.32	41.92	34.04	18.32	20.06	5.85	56.95	0.00
**Adaptation ratio**	0.48	0.14	0.63	0.11	0.60	0.08	0.49	0.00
**Bursting (IEI)**	101.86	100.36	145.61	209.55	205.37	140.80	50.00	0.00
**Rebound ratio**	31.79	21.52	65.68	49.52	33.95	8.78	27.00	0.00

Table S3. Electrophysiological parameters of PatchSeq analyzed cells. Related to Figure 5.

Table showing electrophysiological parameters used in PCA (Figure 5C-E). Mean ± SD is presented for each assigned molecular group (Pthlh, Th, Npy/Mia and Cck/Vip). For each cluster the number of cells analyzed is indicated. SD: standard deviation, RMP: resting membrane potential, AP: action potential, AHP: after-hyperpolarization.

	Layer V, S1 cortex (20 cells)		Dorsal striatum (25 cells)				
	**Mean**	**SD**	**Mean**	**SD**	**p-value**	**q-value**	**Sig**
**Age**	28.38	5.05	28.96	2.15	0.6054	0.8289	ns
**Input resistance (MΩ)**	165.57	49.31	122.16	71.03	0.0227	0.0499	*
**RMP (mV)**	-68.67	7.23	-69.88	5.10	0.5092	0.7713	ns
**Time constant (τ)**	7.97	3.24	3.61	1.65	8.67E-06	0.0001	***
**Rheobase (pA)**	138.90	68.32	249.68	114.55	0.0003	0.0012	**
**AP treshold (mV)**	-38.52	7.15	-35.41	5.43	0.1016	0.1839	ns
**AP amplitude (mV)**	61.57	11.88	58.11	10.68	0.304	0.2689	ns
**Half width (ms)**	0.67	0.21	0.63	0.24	0.5626	0.6902	ns
**AHP amplitude (mV)**	17.17	3.46	13.97	3.05	0.0018	0.0012	**
**AHP latency (ms)**	2.68	0.92	4.37	3.04	0.0184	0.0358	*
**Latency for first AP (ms)**	127.02	206.08	418.40	280.07	0.0003	0.0012	**
**AP repolarization latency (ms)**	0.05	0.03	0.06	0.09	0.564	0.6761	ns
**Sag ratio**	-0.02	0.01	-0.01	0.01	0.0349	0.0527	ns
**Max frequency (Hz)**	207.98	73.30	184.80	87.45	0.3408	0.2930	ns
**Steady frequency (Hz)**	140.85	71.38	98.60	54.79	0.02382	0.0408	*
**Adaptation ratio**	0.66	0.21	0.53	0.13	0.0137	0.0358	*

Table S5. Differences in electrophysiological parameters of striatal and cortical *Pvalb*$^{cre+}$ cells (Unpaired t-tests). Related to figure 7.

Table showing parameters used in PCA (Figure 7C,D) and unpaired t-tests (Figure 7E). Mean ± SD, p-value, q-value (adjusted p-value after Benjamini-Hochberg correction for multiple testing) and t statistics is presented of *Pvalb*$^{cre+}$ cells recorded in dorsal striatum (25) and Layer V Somatosensory cortex (S1; 20 cells). SD: standard deviation, RMP: resting membrane potential, AP: action potential, AHP: after-hyperpolarization.
